# Diagnosis of human leptospirosis: systematic review and meta-analysis of the diagnostic accuracy of the *Leptospira* microscopic agglutination test, PCR targeting *Lfb1*, and IgM ELISA to *Leptospira fainei* serovar Hurstbridge

**DOI:** 10.1186/s12879-023-08935-0

**Published:** 2024-02-07

**Authors:** Marta Valente, Justina Bramugy, Suzanne H. Keddie, Heidi Hopkins, Quique Bassat, Oliver Baerenbold, John Bradley, Jane Falconer, Ruth H. Keogh, Paul N. Newton, Mathieu Picardeau, John A. Crump

**Affiliations:** 1 ISGlobal, Hospital Clínic - Universitat de Barcelona, Calle Rosselló, 171, Entresol, Barcelona, 08036 Spain; 2 Centro de Investigação em Saúde de Manhiça (CISM), Maputo, Mozambique; 3 London School of Hygiene & Tropical Medicine, London, UK; 4 ICREA, Pg. Lluís Companys 23, Barcelona, 08010 Spain; 5 Pediatrics Department, Hospital Sant Joan de Déu, Universitat de Barcelona, Esplugues, Barcelona, Spain; 6 Consorcio de Investigación Biomédica en Red de Epidemiología y Salud Pública (CIBERESP), Madrid, Spain; 7 MRC International Statistics and Epidemiology Group, London School of Hygiene and Tropical Medicine, London, UK; 8 Centre for Tropical Medicine and Global Health, Nuffield Department of Medicine, University of Oxford, Oxford, UK; 9 Lao-Oxford-Mahosot Hospital Wellcome Trust Research Unit, Microbiology Laboratory, Mahosot Hospital, Vientiane, Laos; 10 Biology of Spirochetes Unit, French National Reference Centre for Leptospirosis, Institut Pasteur, Université Paris Cité, Paris, F-75015 France; 11 Centre for International Health, University of Otago, Dunedin, New Zealand

**Keywords:** Leptospirosis, Meta-analysis, Agglutinations tests, Polymerase chain reaction, Enzyme-linked immunosorbent assay, Systematic review, Sensitivity and specificity

## Abstract

**Background:**

Leptospirosis is an underdiagnosed infectious disease with non-specific clinical presentation that requires laboratory confirmation for diagnosis. The serologic reference standard remains the microscopic agglutination test (MAT) on paired serum samples. However, reported estimates of MAT’s sensitivity vary. We evaluated the accuracy of four index tests, MAT on paired samples as well as alternative standards for leptospirosis diagnosis: MAT on single acute-phase samples, polymerase chain reaction (PCR) with the target gene *Lfb1*, and ELISA IgM with *Leptospira fainei* serovar Hurstbridge as an antigen.

**Methods:**

We performed a systematic review of studies reporting results of leptospirosis diagnostic tests. We searched eight electronic databases and selected studies that tested human blood samples and compared index tests with blood culture and/or PCR and/or MAT (comparator tests). For MAT selection criteria we defined a threshold for single acute-phase samples according to a national classification of leptospirosis endemicity. We used a Bayesian random-effect meta-analysis to estimate the sensitivity and specificity of MAT in single acute-phase and paired samples separately, and assessed risk of bias using the Quality Assessment of Studies of Diagnostic Accuracy Approach- 2 (QUADAS-2) tool.

**Results:**

For the MAT accuracy evaluation, 15 studies were included, 11 with single acute-phase serum, and 12 with paired sera. Two included studies used PCR targeting the *Lfb1* gene, and one included study used IgM ELISA with *Leptospira fainei* serovar Hurstbridge as antigen. For MAT in single acute-phase samples, the pooled sensitivity and specificity were 14% (95% credible interval [CrI] 3–38%) and 86% (95% CrI 59–96%), respectively, and the predicted sensitivity and specificity were 14% (95% CrI 0–90%) and 86% (95% CrI 9–100%). Among paired MAT samples, the pooled sensitivity and specificity were 68% (95% CrI 32–92%) and 75% (95% CrI 45–93%) respectively, and the predicted sensitivity and specificity were 69% (95% CrI 2–100%) and 75% (2–100%).

**Conclusions:**

Based on our analysis, the accuracy of MAT in paired samples was not high, but it remains the reference standard until a more accurate diagnostic test is developed. Future studies that include larger numbers of participants with paired samples will improve the certainty of accuracy estimates.

**Supplementary Information:**

The online version contains supplementary material available at 10.1186/s12879-023-08935-0.

## Background

Leptospirosis is an underdiagnosed infectious disease, with an estimated global annual number of illnesses of more than one million per year from 1970 to 2008 [1], 60,000 estimated annual deaths [1], and a mortality ratio ranging from 2% through to 60%, among older patients with icteric disease or renal failure [2]. Although tropical regions have the highest incidence of disease, with climate change and massive urbanization of frequently flooded areas in low-income countries, the epidemiology of this zoonosis is changing and it is a growing global public health problem [3–5]. In tropical and subtropical settings, the symptoms and signs of leptospirosis overlap with those of many other acute febrile illnesses including malaria, arboviral, and rickettsial diseases, and thus require laboratory confirmation for diagnosis [6–8].

Numerous diagnostic tests based on nucleic acid or antibody detection have been developed for early diagnosis of leptospirosis [9], but the serologic reference standard remains the microscopic agglutination test (MAT) on paired samples with a four-fold or greater rise, or seroconversion, confirming the diagnosis [10, 11]. Nevertheless, reported estimates of sensitivity vary [12, 13]. The clinical characteristics of the populations studied, including days post-onset of symptoms and prior use of antibacterials, the serovars included in the MAT panel in relation to the epidemiology of the disease in the geographic region studied, as well as the laboratory performance, contribute to heterogeneous estimates of MAT sensitivity in paired samples [11–13].

Because MAT is an imperfect reference test, accuracy evaluations that do not account for the imperfect nature of the test are biased [13, 14]. To explore this, Bayesian latent class analysis can be used to estimate the accuracy of a test, without assuming that any test is 100% accurate [15]. To our knowledge there is no published systematic review regarding MAT diagnostic accuracy using latent class analysis.

The Febrile Illness Evaluation in a Broad Range of Endemicities (FIEBRE) study is a prospective observational study of the infectious causes of fever at four sites in Africa and Asia, collecting data and samples from adult and paediatric outpatients, inpatients, and community controls [16]. FIEBRE tests for preventable and treatable infections, including leptospirosis, using reference standard diagnostic tests performed at specialised laboratory centres of excellence. The approach for the diagnosis of leptospirosis used in FIEBRE was an initial IgM ELISA screen using *Leptospira fainei* serovar Hurstbridge antigen on participants’ convalescent sera, or for participants who did not provide convalescent serum, screening of acute serum from the day of clinical presentation. For IgM ELISA positive samples, MAT using a globally representative panel of *Leptospira* serovars enriched when possible with local strains was performed on acute and, when available, convalescent sera. MAT was also performed on all acute plasma samples positive by SYBR Green based real-time polymerase chain reaction (PCR) assay targeting the *Lfb1* gene [17, 18].

We conducted a systematic review and meta-analysis to assess the accuracy of the index tests: MAT, PCR with the pathogenic *Leptospira* target gene *Lfb*1, and ELISA IgM with the target antigen *Leptospira fainei* serovar Hurstbridge. We compared the index tests with reference standard diagnostic tests for lepstospirosis diagnosis [10]: blood culture and/or PCR and/or MAT (comparator tests). We used a Bayesian latent class model to evaluate the sensitivity and specificity of MAT on single acute-phase samples and MAT on paired samples.

## Methods

### PROSPERO protocol

The protocol of our systematic review was developed prior to conducting the review, and was registered in the International Prospective Register of Systematic Reviews (PROSPERO) at https://www.crd.york.ac.uk/PROSPERO/display_record.php?RecordID=285773, registration number CRD42021285773.

### Search strategy

The original searches were conducted by a library information specialist (JF) on 9 September 2020 for PCR, 10 September 2020 for MAT, and 30 November 2020 for IgM ELISA, and all searches were updated on 16 August 2022. Databases searched included OvidSP Medline, OvidSP Embase, OvidSP Global Health, Wiley Cochrane Central Register of Controlled Trials, Clarivate Analytics Web of Science (Science Citation Index Expanded and Social Sciences Citation Index only), Elsevier Scopus, Ebsco Africa-Wide Information, World Health Organization (WHO) Latin American and Caribbean Health Sciences Literature, and WHO Global Index Medicus.

The search included strings of terms, synonyms, and controlled vocabulary terms to reflect two concepts: leptospirosis, and either MAT, PCR, or IgM ELISA, hereafter referred to as the index test of each search. The exact search terms used for each search are shown in the Supplementary material (Appendix S1). Animal studies were excluded, and the search was limited by date of publication from 1950 when MAT protocols were initially published [19] through 16 August 2022. Duplicates were removed. Additional eligible studies were found by manually searching the reference lists of relevant manuscripts and by contacting authors.

### Selection criteria

The selection criteria applied to all studies found in the search are detailed in Table 1.

**Table 1:** Selection criteria applied to studies found in the systematic review of studies evaluating the diagnostic accuracy of MAT, PCR, and IgM ELISA, published global and between 1950–2022

Selection criteria
1) Studies performed using human blood samples
2) Observational and interventional studies among patients with fever history or suspected leptospirosis
3) Article in English, Spanish or Portuguese
4) Test of interest (MAT, PCR targeting the *Lfb1* gene or IgM ELISA with the target antigen Hurstbridge) and at least one comparator test (MAT, PCR with any target gene or Culture) performed on the same samples
5) Data for extraction of a 2 × 2 contingency table
6) For studies for MAT accuracy evaluation, results of testing single acute samples presented separately from results of testing paired samples (i.e. acute and convalescent samples)
7) For studies for MAT accuracy evaluation, threshold for single acute-phase samples in endemic settings ≥ 1:400 and in non-endemic setting ≥ 1:100

For the MAT systematic review, we included the threshold of single acute-phase sample in the selection criteria. Since leptospirosis case definitions for single acute-phase samples vary according to background seroprevalence [10], we sub-classified the study settings considering where leptospirosis is endemic and non-endemic based on national level assessments. In line with Costa et al. [1] we considered non-endemic settings to be countries with 10 or fewer leptospirosis cases per 100,000 population per year, and endemic settings to be countries with more than 10 cases per 100,000 population per year. Costa’s review [1] identified 80 studies from 34 countries that fulfilled the selection and quality criteria for a disease incidence study with a defined study period of leptospirosis endemic transmission, and developed a multivariable regression model to estimate leptospirosis incidence for each country and territory.

Following this rationale, we set as selection criteria the titre cut-off for a positive MAT in a single acute-phase sample of ≥ 1:400 for endemic settings, and ≥ 1:100 for non-endemic settings. For all settings, the criteria for a serologically confirmed case of leptospirosis was defined as seroconversion or a four-fold or greater rise in MAT antibody titre between paired samples from a person with a history of measured or reported fever, or with suspected leptospirosis [10].

### Study selection and data extraction

Two reviewers (JB, MV) screened and selected all studies independently and in duplicate, using two separate Excel spreadsheets (Authors, Title, Abstract, Journal, Year, Volume, Issue, Pages, DOI) for MAT and PCR studies, and for IgM ELISA studies using the online tool Cadima (https://www.cadima.info/) [20].

The initial eligibility assessment of all titles and abstracts identified by the search strategy was performed using the predetermined selection criteria (Table 1). Full-text copies of all potentially eligible reports were retrieved and reviewed, independently and in duplicate by JB and MV. Any disagreements about eligibility were resolved through discussion between JB and MV, leading to the inclusion of reports meeting all selection criteria and exclusion of those not meeting criteria. For each included report, JB and MV independently abstracted data using a standardized data abstraction sheet that was first piloted on fifteen studies (see Supplementary material, Table S1). We contacted study investigators when a report appeared to meet selection criteria, but data reported were unclear or insufficient to abstract a 2 × 2 contingency table comparing one or more index with another test. If sufficient data were not available or there was no reply from the authors, the study was excluded.

### Bias assessment

We assessed study quality using the revised Quality Assessment of Diagnostic Accuracy Studies (QUADAS-2) criteria, which assesses both the risk of bias and applicability to the review question for four domains: participant selection, index test, reference standard, and flow and timing of participants [21]. Each included article was graded as ‘low risk’ or ‘high risk.’ Each category was defined according to the criteria included in the manuscript, as shown in Tables 2 and 3.

**Table 2:** Criteria for assessing bias in the systematic review of studies evaluating the diagnostic accuracy of MAT, PCR, and IgM ELISA, published global and between 1950–2022

Domain	Grade	Criteria
A. Criteria for assessing bias in studies selected for MAT accuracy evaluation		
Patient selection	Low risk	Prospective studies and case-control studies in the same population
	High risk	Case-control studies in different populations or healthy controls; eligibility other than suspected leptospirosis
Index test (MAT)	Low risk	MAT performed in paired samples with a positivity criteria of ≥ 4-fold rise or seroconversion
	High risk	MAT performed in single acute-phase samples; any other positivity criteria for paired samples different than ≥ 4-fold rise or seroconversion
Comparator test (culture and/or PCR)	Low risk	Performed in recruitment samples; performed according to standard methodology
	High risk	Performed in convalescent samples; not performed according to standard methodology
Flow and timing	Low risk	All patients subject to the same comparator tests; comparator tests and index test performed on samples taken at the same time for acute phase
	High risk	Not all participants performed the same comparator test; use of samples collected on different days for acute phase
B. Criteria for assessing bias in studies selected for PCR accuracy evaluation		
Patient selection	Low risk	Prospective studies and case-control studies in the same population
	High risk	Case-control studies in different populations or healthy controls; eligibility other than suspected leptospirosis
Index test (PCR)	Low risk	Performed in recruitment samples; performed according to standard methodology
	High risk	Performed in convalescent samples; not performed according to standard methodology
Comparator test (MAT and/or culture and/or PCR)	Low risk	Use of MAT on paired samples in at least 75% of participants; cases defined with ≥ 4-fold rise in antibody titers or with a positive culture of Leptospira; tests performed according to standard methodology
	High risk	Use MAT on less than 75% of paired samples, any other positivity criteria for paired samples different than ≥ 4-fold rise or seroconversion; tests not performed according to described methodology
Flow and timing	Low risk	All patients subject to the same comparator tests; comparator tests and index tests performed on samples collected at the same time for acute phase
	High risk	Not all participants performed the same comparator test; use of samples collected on different days for acute phase
C. Criteria for assessing bias in studies selected for IgM ELISA accuracy evaluation		
Patient selection	Low risk	Prospective studies and case-control studies in the same population
	High risk	Case-control studies in different populations or healthy controls; eligibility other than suspected leptospirosis
Index test (IgM ELISA)	Low risk	Threshold for positivity defined a priori; test performed according to manufacturer’s recommendations
	High risk	Threshold for positivity not defined a priori; test not performed according to manufacturer’s recommendations
Comparator test (MAT, culture and/or PCR)	Low risk	Use of MAT on paired samples in at least 75% of participants, cases defined as a positive PCR, MAT with ≥ 4-fold rise in antibody titers or a positive culture of *Leptospira*; tests performed according to described methodology
	High risk	Use MAT on less than 75% of paired samples; culture and PCR performed in convalescent samples, any other positivity criteria for MAT than ≥ 4-fold rise or seroconversion between paired samples; tests not performed according to standard methodology
Flow and timing	Low risk	All patients subject to the same comparator tests; comparator tests and index test performed on samples collected at the same time for acute phase
	High risk	Not all participants performed the same comparator test; use of samples collected on different days for acute phase

**Table 3:** Criteria for assessing applicability in the systematic review of studies evaluating the diagnostic accuracy of MAT, PCR, and IgM ELISA, published global and between 1950–2022

Domain	Grade	Criteria
A. Criteria for assessing applicability in studies selected for MAT accuracy evaluation		
Patient selection	Low risk	Patients with a febrile illness, symptoms of leptospirosis or fever of unspecified duration
	High risk	Patients without febrile illness or without clinical suspicious of leptospirosis
Index test (MAT)	Low risk	Panel of local known circulating serovars; where local serovars are unknown, a globally representative serovar panel is used; MAT performed according to described methodology
	High risk	Panel without local circulating serovars; MAT not performed according to described methodology
Comparator test (Culture and/or PCR)	Low risk	PCR and/or culture performed according to standard methodology
	High risk	PCR and/or culture not performed according to standard methodology
B. Criteria for assessing applicability in studies selected for PCR accuracy evaluation		
Patient selection	Low risk	Patients with febrile illness, symptoms of leptospirosis or fever of unspecified duration
	High risk	Patients without febrile illness or without clinical suspicious of leptospirosis
Index test (PCR)	Low risk	PCR performed according to standard methodology
	High risk	PCR not performed according to standard methodology
Comparator test (MAT and/or culture and/or PCR)	Low risk	Panel of local known circulating serovars; where local serovars are unknown, a globally representative serovar panel is used; tests performed according to standard methodology
	High risk	Panel without local circulating serovars; tests not performed according to standard methodology
C. Criteria for assessing applicability in studies selected for IgM ELISA accuracy evaluation		
Patient selection	Low risk	Patients with febrile illness, symptoms of leptospirosis or fever of unspecified duration
	High risk	Patients without febrile illness or without clinical suspicious of leptospirosis
Index test (IgM ELISA)	Low risk	IgM ELISA performed according to standard methodology
	High risk	IgM ELISA not performed according to standard methodology
Comparator test (MAT, culture and/or PCR)	Low risk	Panel of local known circulating serovars; where local serovars are unknown, a globally representative serovar panel is used; MAT, PCR and/or culture performed according to standard methodology
	High risk	Panel without local circulating serovars; MAT, PCR and/or culture not performed according to standard methodology

### Data analysis

For analysis we required data from each study in the form of a 2 × 2 contingency table showing results of the index test and a comparator test. The index test was any of the tests of interest for each systematic review: single acute-phase MAT, paired MAT, PCR with target gene *Lfb1,* or ELISA IgM with target antigen Hurstbridge. The comparator tests were pre-determined before beginning the review according to the reference standard diagnostic tests for lepstospirosis diagnosis [10]. When MAT (on either a single sample or paired sera) was the index test, the comparator tests were blood culture and/or PCR to any target gene; when PCR with target gene *Lfb1* was the index test, the comparator test was MAT (on either a single sample or paired sera) and/or blood culture and/or PCR (with other target genes); when ELISA IgM was the index test, the comparator test was MAT (on either a single sample or paired sera) and/or PCR (with any target gene) and/or blood culture.

Regarding MAT (on either a single sample or paired sera) meta-analysis, when a study reported data on multiple comparator tests, we created separate 2 × 2 contingency tables comparing the index test with each comparator test. In these cases, without individual level data we were unable to include all data in the meta-analyses without introducing bias. To systematically ensure only one 2 × 2 table from each study was included in the meta-analyses, we chose to include the 2 × 2 table where the comparator test was blood culture. This choice was made because more accuracy data on the specificity of blood culture are available than data on the sensitivity or specificity of PCR [22].

We implemented a Bayesian random-effect latent class meta-analysis, which is an extension to the Hierarchical Summary Receiver Operating Characteristic (HSROC) Model [18] to estimate the sensitivity and specificity of index tests. This framework took into account the imperfect nature of all tests included, as well as accounting for within- and between-study variability.

We fitted separate meta-analyses for MAT single acute-phase and paired sera, and for each analysis calculated the median and 95% credible interval (CrI) for the estimated sensitivity and specificity of the index test in each study. Importantly, we also calculated both the estimated median and 95% CrI for sensitivity and specificity across studies, known as pooled accuracy, as well as the predicted sensitivity and specificity. These predicted values estimate the sensitivity and specificity that would be expected if the test were to be used in a hypothetical future study. These pooled and predicted estimates of accuracy are presented through summary Receiver Operating Characteristic (ROC) curves which represent the 95% credible region for the joint estimate of the index tests sensitivity and specificity. If a meta-analysis could not be performed due to scarcity of data, as was the case with PCR and ELISA reviews, we estimated accuracy of the index test in individual studies using latent class analysis [23].

All analyses were carried out in R using stan [24]. A full model specification including sensitivity analysis investigating the impact on estimates of accounting for conditional dependence between tests within a disease class, as well as results where non-endemic studies are excluded, can be found in Supplementary material (Appendix 2). All code can be found at: https://github.com/shk313/diagnostic-test-metaanalysis/tree/main/Leptospirosis.

## Results

### Study selection

#### Single acute-phase and paired MAT

**Fig. 1:**
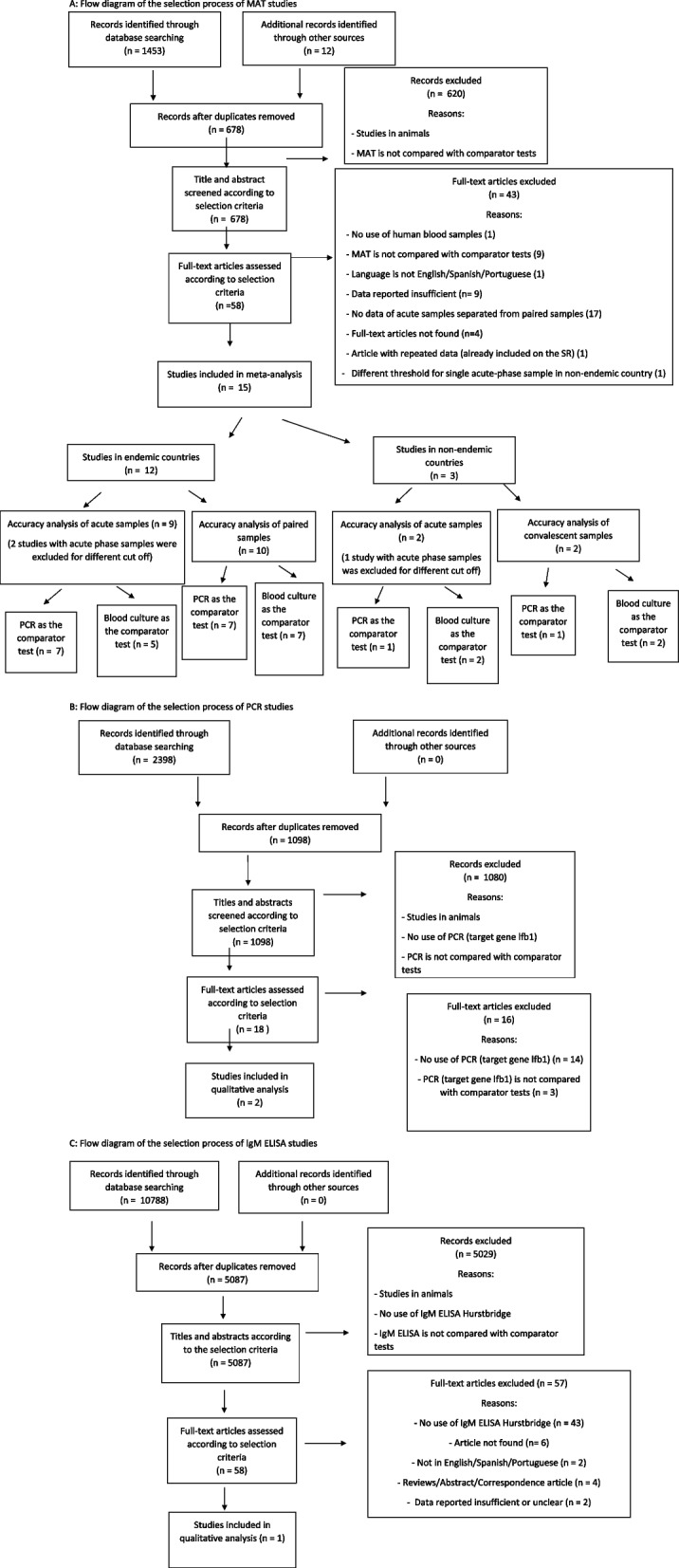
Study flow diagram for systematic review of studies evaluating the diagnostic accuracy of MAT, PCR, and IgM ELISA, published global and between 1950–2022. **A** Flow diagram of the selection process of MAT studies. **B** Flow diagram of the selection process of PCR studies. **C** Flow diagram of the selection process of IgM ELISA studies

Our systematic review of MAT performed on single acute-phase and paired samples identified 691 reports. Of these, 58 (8.4%) were identified as potentially relevant on the basis of the title and abstract and underwent full-text review. Of these, 15 (25.9%) met our selection criteria and were included [25–39]; 12 (80%) [25–36] tested samples from endemic countries and three (20%) [37–39] from non-endemic countries. Of the 12 studies in endemic countries, nine studies (75%) [25–30, 35, 36] reported data from single acute-phase samples and ten studies (83,3%) [25–29, 31–34] reported data from paired samples. Of the three studies in non-endemic countries, two (66.6%) [37, 38] reported data from single acute-phase samples and two (66.6%) [38, 39] from paired samples. We excluded results of single acute-phase samples from three studies [32, 33, 39] because the threshold of detection used was different from our national leptospirosis endemicity-based selection criteria (Fig. 1).

The studies that were not included due having insufficient data available to create a 2 × 2 contingent table for single acute-phase samples and/or paired samples are detailed in Appendix S3.

#### PCR target gene *lfb1*

Our PCR review identified 1,094 reports. Of these, 18 (1.6%) were identified as potentially relevant on the basis of the title and abstract and underwent full-text review. Of these 18 reports, two (11.1%) articles [27, 40] met our selection criteria and were included (Fig. 1).

#### ELISA IgM target antigen *Leptospira fainei* serovar Hurstbridge

Our IgM ELISA review identified 5,092 reports. Of these, 58 (1.1%) were identified as potentially relevant on the basis of title and abstract and underwent full-text review. Of these 58 reports, one (1.7%) article [41] met our selection criteria and was included (Fig. 1).

### Study characteristics

#### Single acute-phase and paired MAT

The characteristics of all included studies are detailed in Table 4. The 15 studies included for MAT (11 (73%) studies were of single-sample MAT, 12 (80%) studies of paired MAT and 8 (53%) studies were of both) were conducted from 2000 through 2020. Of these studies, 14 (93%) of 15 [25–38] included participants with suspected leptospirosis and one (7%) of 15 [39] included participants with fever. Of studies from endemic regions, recruitment occurred in Brazil [28, 29]; Japan [34]; Pacific Island Countries and Territories such as Marquesas Islands, Society Islands, Wallis and Futuna, and New Caledonia [27]; India [32, 33]; Laos [25, 28]; Malaysia [30, 35]; and Thailand [31, 36]. In non-endemic countries, recruitment occurred in New Zealand [39] and Slovenia [37, 38]. All studies were prospective. The MAT panel comprised 20 to 22 serovars in five studies [25, 26, 29, 30, 35], 13 to 15 serovars in three studies [34, 37, 38], and 8 to 11 serovars in three studies [32, 33, 39]. The MAT panel was not described in four studies [27, 28, 31, 36]. The comparator test was blood culture in five studies [29, 32, 33, 36, 37], PCR in four studies [26, 27, 30, 35], and both were used as comparators in six studies [25, 28, 31, 34, 38, 39]. Of studies with PCR as a comparator test, three studies used serum samples [26–28], five used whole blood samples [31, 34, 35, 38, 39], one used both [30], and one study used serum and buffy coat [25]. Recruitment of individuals varied in relation to time of illness onset across studies. The number of days post-onset (DPO) of symptoms at recruitment were 0 to 14 days [34], 1 to 30 days [25, 27], a mean of 6 days [29], and an interquartile range of 2 to 5 [36], 2 to 6 [31], and 3 to 7 days [28]. The DPO of symptoms was not detailed in eight studies [26, 30, 32, 33, 35, 37–39]. The number of days between acute and convalescent samples also varied with reported timeframes including: 7 to 15 days [25, 31, 32], more than 15 days [29, 35, 38], and was not detailed in nine studies [26–28, 30, 33, 34, 36, 37, 39].

**Table 4:** Characteristics of studies selected in the systematic review of studies evaluating the diagnostic accuracy of MAT, PCR, and IgM ELISA, published global and between 1950 – 2022 for MAT, PCR and IgM accuracy evaluation

A. MAT endemic studies																
**Study first author (ref)**	**Title**	**Journal**	**Year of recruitment**	**Country**	**Endemicity**	**Study setting**	**Type of study**	**Participants included**	**Mean age / Children**	**Case definition**	**MAT panel**	**Comparator test**	**DPO of fever at recruitment**	**Sample type for PCR test**	**Acute sample data/Convalescent samples data**	**Interval between acute and convalescent samples**
Woods K [25]	A comparison of two molecular methods for diagnosing leptospirosis from three different sample types in patients presenting with fever in Laos	Clin Microbiol Infect	2014	Vietiane, Laos	Yes	Mahosot Hospital	Prospective	Suspected leptospirosis	39 years (0.5–97) / Yes	Titers of ≥ 1:400 or a fourfold rise in titre	22 serovars. Performed at the WHO/FAO/OIE Collaborating Centre for Leptospirosis Reference and Research, Queensland, Australia	Blood culture and PCR	1–30 days	Serum or buffy coat	Yes/Yes	10–14 days
Blanco R [26]	Evaluation of nested polymerase chain reaction for the early detection of Leptospira spp. DNA in serum samples from patients with leptospirosis	Diagn Microbiol Infect Dis	2010	Brazil, Sao Paulo	Yes	Not stated	Prospective	Suspected leptospirosis	Not stated	Threshold: ≥ 1:800 or a fourfold rise	21 serovars most frequently found in São Paulo, Brazil	PCR	Not stated	Serum	Yes/Yes	Not stated
Merien F [27]	A rapid and quantitative method for the detection of Leptospira species in human leptospirosis	FEMS Microbiol Lett	2004–2005	Pacific Island Countries and Territories	Yes	Not stated	Prospective	Suspected leptospirosis	30 years / Yes	Threshold: ≥ 1:400 or a fourfold rise	Not described	PCR	1–30 days	Serum	Yes/Yes	Not stated
Dittrich S [28]	A Prospective Hospital Study to Evaluate the Diagnostic Accuracy of Rapid Diagnostic Tests for the Early Detection of Leptospirosis in Laos	Am J Trop Med Hyg	2014–2015	Laos	Yes	Mahosot Hospital	Prospective	Suspected leptospirosis or typhus	39 years (0.5–92) / Yes	Threshold: ≥ 1:400 or fourfold rise	MAT was performed and interpreted by the WHO Collaborating Center for Reference and Research on Leptospirosis, Australia	Blood culture and PCR	Interquartile range: 3–7 days	Serum	Yes/Yes	Not stated
Albuquerque A [29]	Validation of a case definition for leptospirosis diagnosis in patients with acute severe febrile disease admitted in reference hospitals at the State of Pernambuco, Brazil	Rev Soc Bras Med Trop	2009	Pernambuco, Brazil	Yes	Hospital Barão de Lucena and Hospital Universitário Oswaldo Cruz	Prospective	Suspected leptospirosis	32.9 (Standard deviation 13.2) / No	Threshold: > 1:800 or a fourfold rise	22 serovars	Blood culture	6.1 ± 2.6 DPO days	Not applicable	Yes/Yes	≥ 14 days
Philip N [30]	Combined PCR and MAT improves the early diagnosis of the biphasic illness leptospirosis	PLoS One	2016–2017	Malasya	Yes	Hospital Serdang, Hospital Tengku Ampuan Rahimah and Hospital Teluk Intan	Prospective	Suspected leptospirosis	Not stated	Threshold: > 1:400 or a fourfold rise	20 serovars local and internationals	PCR	Not stated	Serum and Whole blood	Yes/Yes	Not stated
Dinhuzen J [31]	A prospective study to evaluate the accuracy of rapid diagnostic tests for diagnosis of human leptospirosis	PLoS Negl Trop Dis	2015–2016	Thailand	Yes	15 hospitals in the Srisaket province	Prospective	Suspected leptospirosis	46 (Standard deviation 17) / No	Threshold: > 1:400 or a fourfold rise	Not described	Blood culture and PCR	Interquartile range 2–6 days	Whole blood	Yes/Yes	7 days
Mullan S [32]	An Important Tool for Early Diagnosis of Leptospirosis Cases	J Clin Diagn Res	2008	India	Yes	New Civil Hospital and peripheral health centre of South Gujarat	Prospective	Suspected leptospirosis	Unclear / No	No	11 serogroups	Blood culture	Not stated	Not applicable	No/Yes	15 days
Vijayachari P [33]	Evaluation of Lepto Dri Dot as a rapid test for the diagnosis of leptospirosis	Epidemiol Infect	2000–2001	India	Yes	3 primary health centres in South Andaman	Prospective	Suspected leptospirosis	Not stated	No	10 serovars commonly encountered in India	Blood culture	Not stated	Not applicable	No/Yes	Not stated
Kakita T [34]	Laboratory diagnostic, epidemiological, and clinical characteristics of human leptospirosis in Okinawa Prefecture, Japan, 2003–2020	PLoS Negl Trop Dis	2003–2020	Japan, Okinawa Prefecture	Yes	Clinics and hospitals in Okinawa Prefecture	Prospective	Suspected leptospirosis	Not stated	No	13 serovar strains of 12 serogroups	Blood culture and PCR	0–14 days	Whole blood	No/Yes	Not stated
Alia S [35]	Diagnostic accuracy of rapid diagnostic tests for the early detection of leptospirosis	J Infect Public Health	2016–2017	Malasya	Yes	Hospital Serdang	Prospective	Suspected leptospirosis	Not stated	Threshold: ≥ 1:400 or a fourfold rise	20 serovars	PCR	Not stated	Whole blood	Yes/No	21–30 days
Sukmark T [36]	Diagnostic accuracy of rapid diagnostic tests for the early detection of leptospirosis	PLoS Negl Trop Dis	2012–2014	Thailand	Yes	11 centers in 8 provinces around Thailand	Prospective	Suspected leptospirosis	Not stated	Threshold: ≥ 1:400 or a fourfold rise	Not described	Blood culture	Interquartile range 2–5 days	Not applicable	Yes/No	Not stated
B. MAT non-endemic studies																
**Study first author (ref)**	**Title**	**Journal**	**Year of recruitment**	**Country**	**Endemicity**	**Study setting**	**Type of study**	**Participants included**	**Mean age / Children included**	**Case definition**	**MAT panel**	**Comparator test**	**DPO of fever at recruitment**	**Sample type for PCR test**	**Acute sample data/Convalescent sample data**	**Days between acute and convalescent sample**
Podgoršek D [37]	Evaluation of real-time PCR targeting the lipL32 gene for diagnosis of Leptospira infection	BMC Microbiology	Not stated	Slovenia	No	Different hospitals in Slovenia	Prospective	Febrile Patients	Not stated	Titers of ≥ 1:100	15 serovars from the geographic area	Blood culture	Not stated	Not stated	Yes/No	Not stated
Podgoršek D [38]	Evaluation of the immunochromatographic (Leptocheck) test for detection of specific antibodies against leptospires	Wien Klin Wochenschr	Not stated	Slovenia	No	Different hospitals in Slovenia	Prospective	Suspected leptospirosis	Not stated	Titers of ≥ 1:100	13 serovars from the geographic area	Blood culture and PCR	No stated	Whole blood	Yes/Yes	14–30 days
Earl L [39]	An evaluation of diagnostic tests in a case series of suspected leptospirosis patients seen in primary care	N Z Med J	Not stated	New Zealand	No	General practices in Waikato and 3 medical centers in Wairoa	Prospective	Suspected for Leptospirosis	39 years (11–73) /Yes	Threshold: > 1:400 andourfold rise	8 serovars	Blood culture and PCR	Not stated	Whole Blood	No/Yes	Not stated
C. PCR studies																
**Study first author (ref)**	**Title**	**Journal**	**Year of recruitment**	**Country**	**Endemicity**	**Study setting**	**Type of study**	**Participants included**	**Mean age / Children included**	**DPO of fever at recruitment**	**Sample type for PCR test**	**Comparator test**	**MAT Case definition for a positive acute sample**	**MAT case definition for a positive convalescent sample**	**MAT Paired samples**	**MAT panel**
Merien F [27]	A rapid and quantitative method for the detection of Leptospira species in human leptospirosis	FEMS Microbiology Letters	2004–2005	Pacific Island Countries and Territories	High	Not stated	Prospective	Suspected leptospirosis	30 years / Yes	1–30 days	Serum	MAT	≥ 1:400 titer	Seroconversion or two fold-rise between titers	10/41	Unclear
Esteves L [40]	Diagnosis of Human Leptospirosis in a Clinical Setting: Real-Time PCR High Resolution Melting Analysis for Detection of Leptospira at the Onset of Disease	Scientific Reports	2015–2016	Azores	High	Hospital	Prospective	Suspected leptospirosis	48,2 years (Standard deviation 16,4) / Not stated	Unclear	Serum	PCR rrs	Not applicable	Not applicable	Not applicable	Not applicable
D. IgM ELISA studies																
**Study first author (ref)**	**Title**	**Journal**	**Year of recruitment**	**Country**	**Endemicity**	**Study setting**	**Type of study**	**Participants included**	**Median age / Children included**	**DPO of fever at recruitment**	**Sample type for IgM ELISA**	**Comparator test**	**MAT Case definition for a positive acute sample**	**MAT case definition for a positive convalescent sample**	**MAT Paired samples**	**MAT panel**
Bourhy P [41]	Evaluation of an in-house ELISA using the intermediate species Leptospira fainei for diagnosis of leptospirosis	Journal of clinical microbiology	Not applicable	Mainland France and French overseas territories	High and low	Hospital	Case–control, with mixed population	Suspected leptospirosis, other diseases, healthy donors	44 years / Yes	Not stated	Serum	MAT	≥ 1:400 titer	Seroconversion	Unclear	22 serogroups

#### PCR target gene *lfb1*

The two studies included for PCR accuracy analysis were conducted 2004–2005 [27] and 2015–2016 [40]. Both studies included patients with suspected leptospirosis, were prospective, and enrolled in the endemic countries Azores [40], and the Pacific Island Countries and Territories of Marquesas Islands, New Caledonia, Society Islands, and Wallis and Futuna [27]. In one study [27] the comparator test was MAT, in which the MAT panel was not described, and 10 (24%) of 41 patients had paired samples. In other study [40] the comparator test was PCR targeting the *rrs* gene in serum samples. The DPO of symptoms was of 1 to 30 days in one study [27] and was not described in other study [40].

#### ELISA IgM with antigen *Leptospira fainei* serovar Hurstbridge

The eligible study included for IgM ELISA accuracy analysis [41] was conducted in France, French Polynesia, Guadeloupe, Guyana, and Martinique, and was a two-gate design study that included patients with suspected leptospirosis and controls from patients with evidence of recent infection for dengue and syphilis, or from healthy blood donors. IgM ELISA was performed in serum samples and the comparator test was MAT. The MAT panel included 22 serovars, and it was not mentioned how many participants had paired samples.

### Study quality

The results of bias assessment are shown in Table 5.

**Table 5:** Bias assessment in the systematic review of studies evaluating the diagnostic accuracy of MAT, PCR, and IgM ELISA, published global and between 1950–2022

A. Endemic countries														
**Author, reference**		**Year**	**Participant Selection**		**Bias**						**Applicability**			
					**Index Test**			**Comparator Test**	**Flow and timing**		**Participant Selection**	**Index Test**		**Comparator Test**
					**Single MAT studies**		**Paired MAT studies**							
Woods K [25]		2017	Low Risk		High Risk		Low Risk	Low Risk	Low Risk		Low Risk	Low Risk		Low Risk
Blanco R [26]		2014	Low Risk		High Risk		Low Risk	Low Risk	Low Risk		Low Risk	Low Risk		Low Risk
Merien F [27]		2005	Low Risk		High Risk		Low Risk	Low Risk	Low Risk		Low Risk	Unclear Risk		Low Risk
Dittrich S [28]		2018	Low Risk		High Risk		Low Risk	Low Risk	Low Risk		Low Risk	Unclear Risk		Low Risk
Albuquerque A [29]		2011	Low Risk		High Risk		Low Risk	Unclear Risk	Low Risk		Low Risk	Low Risk		Unclear Risk
Philip N [30]		2020	Low Risk		High Risk		Low Risk	Low Risk	Low Risk		Low Risk	Low Risk		Low Risk
Dinhuzen J [31]		2021	Low Risk		High Risk		Low Risk	Low Risk	Low Risk		Low Risk	Unclear Risk		Low Risk
Mullan S [32]		2016	Low Risk		NA		Low risk	Low Risk	Low Risk		Low Risk	Low Risk		Low Risk
Vijayachari P [33]		2002	Low Risk		NA		Low risk	Low Risk	Low Risk		Low Risk	Low Risk		Low Risk
Kakita T [34]		2021	Low Risk		NA		Low Risk	Low Risk	Low Risk		Low Risk	High Risk		Low Risk
Alia S [35]		2019	Low Risk		High Risk		NA	Low Risk	Low Risk		Low Risk	Low Risk		Low Risk
Sukmark T [36]		2018	Low Risk		High Risk		NA	Low Risk	Low Risk		Low Risk	Unclear Risk		Low Risk
B. Non-endemic countries														
**Author**		**Year**		**Patient Selection**		**Bias**				**Applicability**				
						**Index Test**		**Comparator Test**	**Flow and timing**	**Patient Selection**			**Index Test**	**Comparator Test**
						**Single MAT studies**	**Paired MAT studies**							
Podgoršek D [37]		2020		Low Risk		High risk	NA	Low Risk	Low Risk	Low Risk			Low Risk	Low Risk
Podgoršek D [38]		2015		Low Risk		Hish risk	Low Risk	Low Risk	Low Risk	Low Risk			Low Risk	Low Risk
Earl L [39]		2021		Low Risk		NA	Low Risk	Low Risk	Low Risk	Low Risk			High Risk	Low Risk
C. Bias assessment of studies selected for PCR accuracy evaluation														
**Author**	**Year**			**Bias**					**Applicability**					
				**Patient Selection**		**Index Test**	**Comparator Test**	**Flow and timing**	**Patient Selection**	**Index Test**			**Comparator Test**	
Merien F [27]	2005			Low Risk		Low Risk	High Risk	Low Risk	Low Risk	Low risk			Unclear Risk	
Esteves L [40]	2011			Low Risk		Low Risk	Low Risk	Low Risk	Low Risk	Low risk			Low Risk	
D. Bias assessment of studies selected for IgM ELISA accuracy evaluation														
**Author**	**Year**			**Bias**					**Applicability**					
				**Patient Selection**		**Index Test**	**Comparator Test**	**Flow and timing**	**Patient Selection**	**Index Test**			**Comparator Test**	
Bourhy P [41]	2013			High Risk		Low Risk	High Risk	Low Risk	High Risk	Low Risk			Low Risk	

#### Single acute-phase and paired MAT

In the patient domain, all studies were graded as low risk of bias and applicability, because they were all prospective and with a population of suspected leptospirosis or febrile patients. In the index test domain, when studies used single acute-phase samples for a confirmatory diagnosis of leptospirosis [25–31, 35–38], they were graded as high risk of bias. When studies used paired samples for a confirmatory diagnosis of leptospirosis [25–34, 38, 39], they were graded low risk of bias on the basis that the positivity criteria included a four-fold rise or greater, or seroconversion, between samples. Regarding applicability, nine studies were graded low risk because they used a globally representative panel of 20 to 22 serovars [25, 26, 29, 30, 35], or used 10 to 15 locally known circulating serovars [32, 33, 37, 38]. Two studies [34, 39] were graded high risk since the MAT panels composed of 13 serogroups and eight serovars, respectively, and they were not mentioned as being locally representative of the study setting. Finally, four studies [27, 28, 31, 36] were graded high risk because MAT panel composition was not described.

In the comparator test domain, regarding bias and applicability, 14 studies [25–28, 30–39] were graded low risk because the comparator tests were performed in recruitment samples and according to standard methodology. One study [29] was graded high risk because laboratory procedures were not described or referenced. For the timing and flow domain, all studies were graded low risk of bias because patients were subject to the same comparator tests, and comparator tests and index test were performed on samples taken at the same time for acute phase.

#### PCR target gene *lfb1*

In the patient and index test domain both PCR studies [27, 40] were graded low risk for quality concerns because they were prospective, in patients suspected of leptospirosis, and the index test was performed in recruitment samples and according to standard methodology. In the comparator test domain, one study [27] was graded high risk of bias because MAT was the comparator test and less than 75% of the samples were paired samples, and graded as high risk for applicability concerns because the MAT panel composition was not described. The second study [40] was graded low risk for quality concerns since the comparator test was performed according to standard methodology. For timing and flow domain, both studies were graded low risk of bias because patients were subject to the same comparator tests, and comparator tests and index test were performed on samples taken at the same time for acute phase.

#### ELISA IgM target antigen *Leptospira fainei* serovar Hurstbridge

The single IgM ELISA study [41] was graded high risk of bias and high risk for applicability concerns in the patient domain, because it was a two-gate design study and controls were healthy blood donors or patients with other diseases. In the index test domain, it was graded low risk for quality concerns since it was performed according to detailed standard methodology and the threshold for positivity defined a priori. In the comparator test domain, it was graded as high risk of bias because MAT was the comparator test and there was no information regarding the use of paired samples for a confirmatory case. For timing and flow domain, it was graded as low risk of bias since patients were subject to the same comparator tests, and comparator tests and index test were performed on samples taken at the same time for acute phase.

### Sensitivity and specificity estimates

#### Single acute-phase and paired MAT

Overall, 11 studies with data on single acute-phase samples representing 2,625 individuals and 12 studies on paired samples representing 1,721 individuals were included in a meta-analysis for MAT. Abstracted data are detailed in Supplementary material, Table S2.

**Fig. 2:**
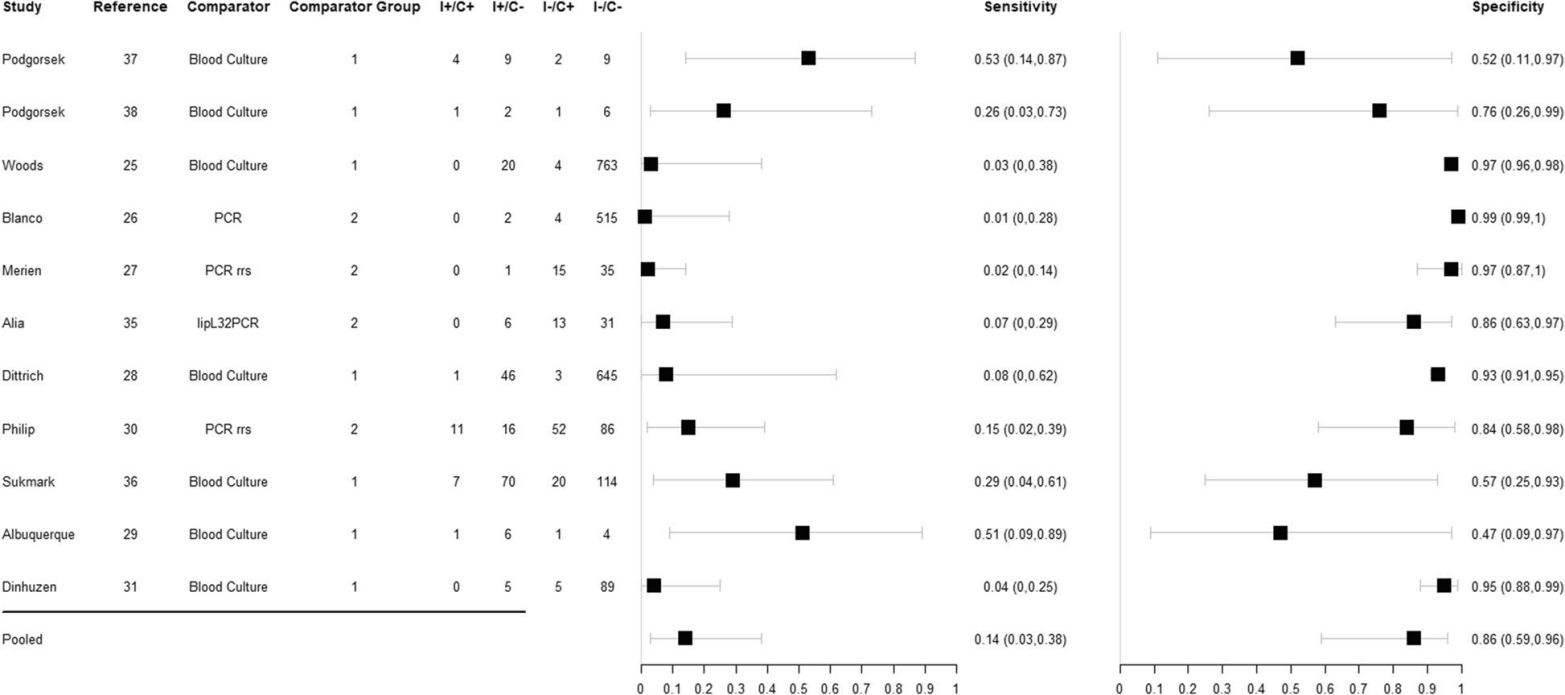
Forest plot of estimated and pooled sensitivity and specificity of studies evaluating the diagnostic accuracy of MAT in single acute-phase samples, published global and between 1950–2022

**Fig. 3:**
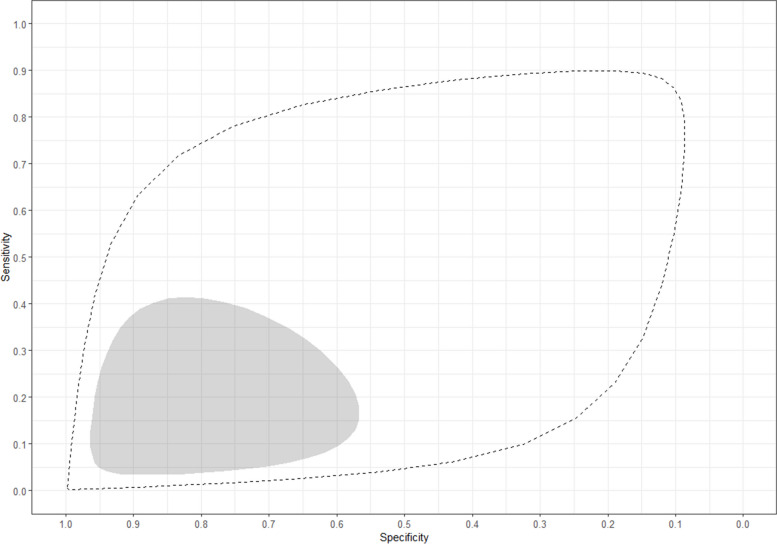
Roc curve of pooled and predicted sensitivity and specificity of studies evaluating the diagnostic accuracy of MAT in single acute-phase samples, published global and between 1950–2022

For single acute-phase samples, the pooled sensitivity and specificity of MAT were 14% (95% CrI 3–38%) and 86% (95% CrI 59–96%), respectively, and the predicted sensitivity and specificity were 14% (95% CrI 0–90%) and 86% (95% CrI 9–100%). The estimates for the sensitivity and specificity of MAT in each individual study can be found in Fig. 2 and the summary receiver operating characteristic (SROC) curves representing the pooled and predicted estimates in Fig. 3.

**Fig. 4:**
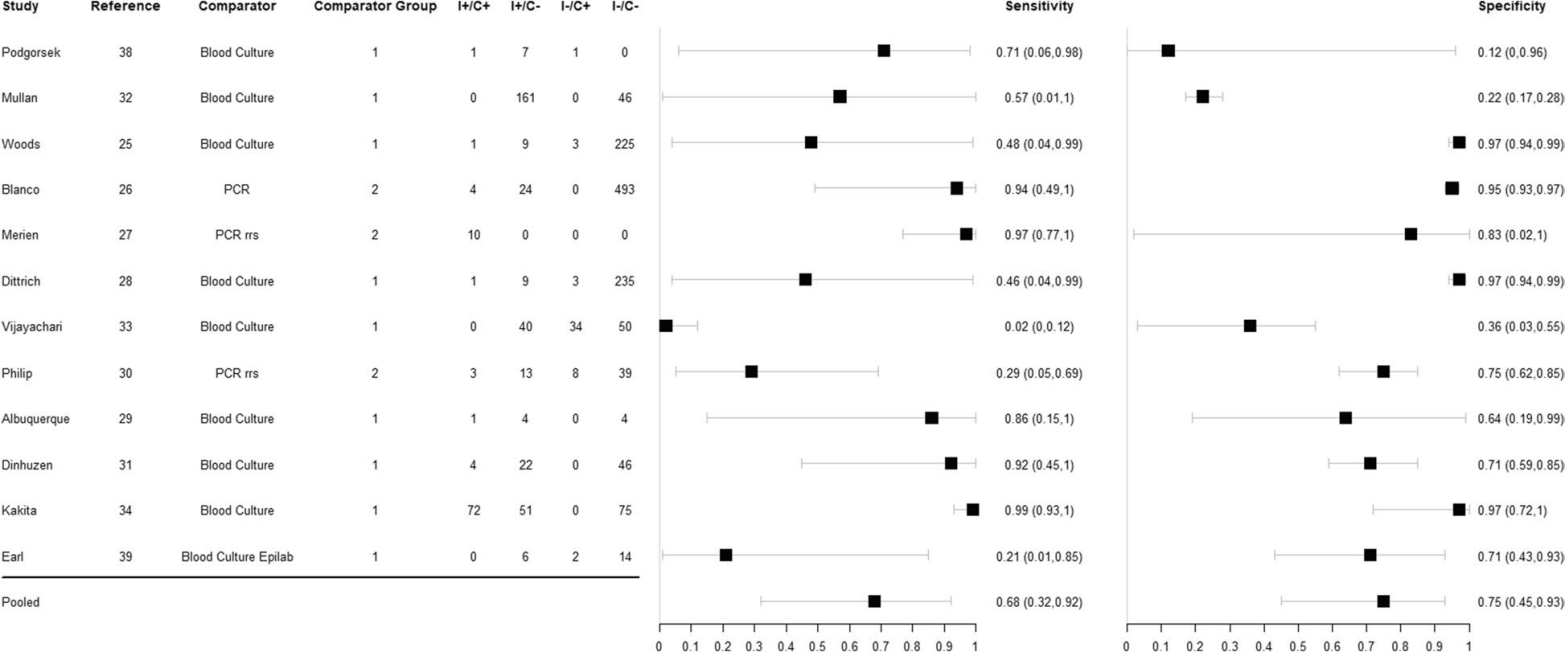
Forest plot of estimated and pooled sensitivity and specificity of studies evaluating the diagnostic accuracy of MAT in paired samples, published global and between 1950–2022

**Fig. 5:**
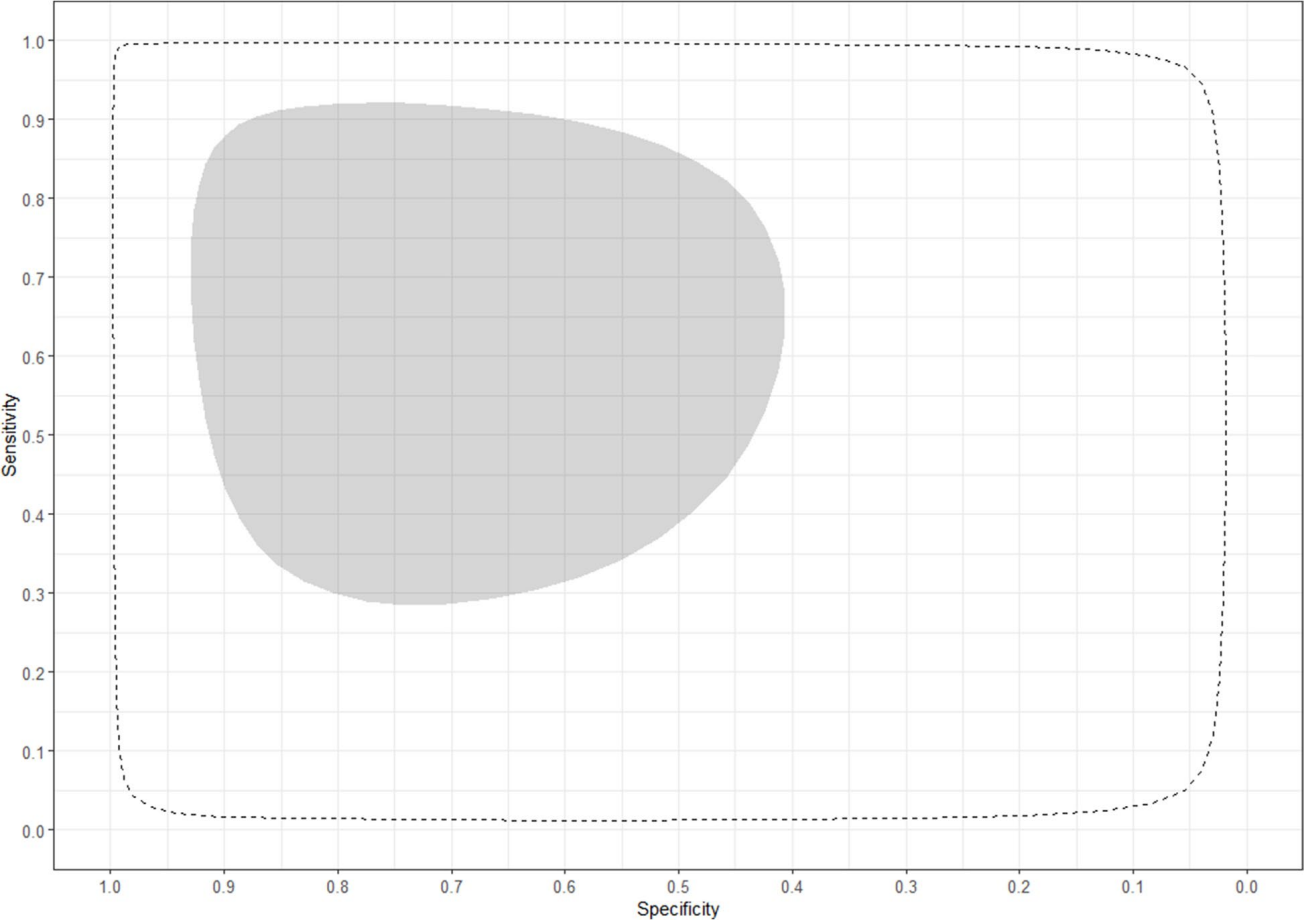
Roc curve of pooled and predicted sensitivity and specificity of studies evaluating the diagnostic accuracy of MAT in paired samples, published global and between 1950–2022

Among paired samples, the pooled sensitivity and specificity of MAT were 68% (95% CrI 32–92%) and 75% (95% CrI 45–93%) respectively, and the predicted sensitivity and specificity were 69% (95% CrI 2–100%) and 75% (95% CrI 2–100%). The estimates for individual studies can be found in Fig. 4 and the SROC curves for pooled and predicted estimates in Fig. 5.

#### PCR targeting *lfb1*

Two studies were included in our review of PCR diagnosis, including a total of 253 individuals. The estimated median sensitivity of PCR in Merien, et al. [27] was 92% (95% CrI 72–100%) and median specificity was 66% (95% CrI 49–91%). In Esteves, et al. [40] the median sensitivity of PCR was 98% (95% CrI 90–100%) and the median specificity was 99% (98–100%) (Table 6).

**Table 6:** Extracted data, sensitivity and specificity estimates in the systematic review of studies evaluating the diagnostic accuracy of PCR and IgM ELISA, published global and between 1950 – 2022

**Study first author, ref**	**Reference test**	**Total N samples**	**Index + /Reference + **	**Index + /Reference-**	**Index-/Reference + **	**Index-/Reference-**	**Sensitivity**	**Specificity**
A. PCR studies								
Merien F [27]	MAT	51	10	15	1	25	92% (72–100)	66% (49–91)
Esteves L [40]	PCR	202	46	0	1	155	98% (90–100)	99% (98–100)
B. IgM ELISA studies								
Bourhy P [41]	MAT	519	298	3	19	199	97% (93–100%)	99% (97–100%)

#### ELISA IgM target antigen *Leptospira fainei* serovar Hurstbridge

A single study that included 519 individuals was identified in our review of IgM ELISA. The estimated median sensitivity of IgM was 97% (93–100%) and the median specificity was 99% (97–100%) (Table 6).

## Discussion

We carried out a systematic review of the sensitivity and specificity of MAT, PCR with the target gene *Lfb1,* and IgM ELISA with the antigen *Leptospira fainei* serovar Hurstbridge for diagnosis of human leptospirosis. Our meta-analysis of 15 studies, including 3,188 participants, found that MAT on single acute-phase samples had a predicted median sensitivity and specificity of 14% and 86%, respectively, for detecting leptospirosis, and using paired samples MAT had a predicted median sensitivity and specificity of 69% and 75%, respectively.

Our estimates of the sensitivity of MAT in single acute-phase samples were low across all studies, but specificity was generally high. These findings are in line with the dynamics of the humoral immune response and with previous work from studies in a variety of countries including the Barbados [42], Netherlands [15], and Sri Lanka [43]. Moreover, numerous studies have shown the value of adding culture, nucleic acid amplification, or antigen detection to MAT serology during the early phase of the disease [44–50].

In paired samples we estimated to correctly identify just over two-thirds of true leptospirosis cases, and correctly reject the diagnosis for three-quarters of suspected cases. We found a more heterogeneous picture of estimated accuracy but our median estimates of 69% sensitivity and 75% specificity were also in line with previous findings in Barbados [42], Brazil [51], and Thailand [52]. Conversely, another study in Thailand [13], that also used a latent class model, estimated sensitivity to be lower than previous studies at 49.9%, with 95% CI from 37.6 to 60.8%. However, the authors stated that this could have been the result of convalescent-phase samples being collected only ten DPO of symptoms, allowing insufficient time for the antibody response to develop, and that 34% of participants did not have convalescent-phase serum specimens collected. Importantly, the estimate of MAT sensitivity in paired samples was 70.3% was consistent with our analysis.

Heterogeneity among studies is reflected in the wide credible intervals for the predicted sensitivity and specificity in this meta-analysis, particularly among the paired samples. The variability in estimates from single acute-phase samples could be explained by the heterogeneity of DPO of fever in the studies included, as shown by Goris et al. [12]. Single acute-phase samples may have been collected early in the illness, less than seven DPO of fever [11], too early in the humoral immune response for it to be a reliably detect infection. The high variability in the sensitivity of MAT in paired samples could be partially explained by the inclusion of patients with a brief interval, less than 14 days [11], between samples, and thus not reaching seroconversion or a four-fold rise or greater between titers [13]. It also could be attributed to failure to consider patients’ use of antimicrobials before testing, particularly relevant when culture was used as a comparator test. It also could be due to MAT panel composition not representing the locally circulating strains [53–55].

Our meta-analysis had several limitations. Firstly, a key assumption of the Bayesian latent class model used is that there exist only two disease classes in the underlying population: diseased and disease-free. If in fact more than two classes exist, this assumption can result in biased estimates of test sensitivity and specificity when conditional independence between tests is assumed [56]. While the results presented in the main text of this paper do not make the assumption of conditional independence between tests, two disease classes are assumed. Further limitations include low geographical diversity, since included studies were conducted in only eight endemic countries, the majority in Southeast Asia, so that our estimates are not representative of all leptospirosis endemic countries. Moreover, our classification of a country’s endemicity followed Costa, et al. [1], but these estimates are based on limited data and do not account for sub-national variation in leptospirosis incidence. Our bias assessment (Table 5) highlights the high risk of bias of all studies using single acute-phase samples as a confirmatory test for leptospirosis, and also that some studies do not describe or account for a globally or locally representative MAT panel, an important quality concern. Moreover, data on DPO of symptoms, the interval between paired samples, and the use of antimicrobials prior to testing were widely heterogeneous or unknown. This information was not included in the quality assessment but could be an important source for bias in some of our studies, interfering with the proportion of positive and negative tests results that correctly identify the infection status of individuals. Also, the low number of positive MAT results in the majority of selected studies compromised power. Another limitation was not finding studies that reported titres on acute and convalescent samples that would have allowed the direct evaluation of single sample MAT in the context of paired MAT. A final limitation was the difficulty in assessing QUADAS-2, due to the lack of detailed data reported on the selected studies and due to the heterogeneity in MAT procedure and panel composition, since laboratories uses diverse antigen panels and every setting has different endemic local *Leptospira* serovars, sometimes unstated.

Our review also has many strengths. To our knowledge, this is the first meta-analysis of MAT accuracy for human leptospirosis diagnosis, and the first using Bayesian latent class modelling to account for the imperfect comparator tests. Our approach took into account different case definitions according to endemicity, and evaluated test results from single acute-phase samples separately from paired samples results. Importantly we used an extensive search strategy, contacted authors for additional data where necessary to complete a 2 × 2 table, and performed in duplicate and independently the process from study screening to data extraction.

Regarding our review of PCR targeting *lfb1* and ELISA IgM targeting antigen *Leptospira fainei* serovar Hurstbridge, due to the scarcity of data available, no meta-analysis could be performed. Instead, we report the estimated accuracy of each test within the included studies only. These results are not generalizable to other studies but suggest that both IgM ELISA and PCR had a high sensitivity in the included studies (median sensitivity: 92%, 98%, and 97%). Specificity varied in the two studies included for PCR (median specificity: 66% and 99%) and was high for IgM ELISA (99%). A 2017 systematic review of IgM ELISA for leptospirosis diagnosis not specifically targeting the antigen *Leptospira fainei* serovar Hurstbridge found similar results [57].

## Conclusions

To our knowledge, this is the first meta-analysis estimating the accuracy of MAT in paired samples for diagnosis of human leptospirosis. Our study found that the sensitivity and specificity of MAT in paired samples were not high. However, MAT on paired sera remains the reference standard until a more accurate diagnostic strategy is developed. A key challenge for our review was the scarcity of high-quality studies driven by a low proportion of participants with paired serum samples, and a lack of detailed reporting of sample timing collection and panel composition. Future studies that use paired samples and that report in detail the sample timing collection and MAT panel composition will improve the certainty of accuracy estimates.

### Supplementary Information

**Additional file 1: Appendix S1.** Search strategy for the systematic review of studies evaluating the diagnostic accuracy of MAT, PCR, and IgM ELISA, published global and between 1950–2022.

**Additional file 2: Appendix S2.** Statistical model the systematic review of studies evaluating the diagnostic accuracy of MAT, PCR, and IgM ELISA, published global and between 1950–2022. **Table S1.** Sensitivity analysis in acute samples. **Table S2.** Sensitivity analysis in convalescent samples.

**Additional file 3: Appendix S3.** List of studies excluded dued to not having enough data available for a 2x2 contingent table for single-acute phase samples and/or paired samples.

**Additional file 4: Table S3.** Standardized extraction sheet form in the systematic review of studies evaluating the diagnostic accuracy of MAT, PCR, and IgM ELISA, published global and between 1950–2022.

**Additional file 5: Table S4.** Extracted data in the systematic review of studies evaluating the diagnostic accuracy of MAT, published global and between 1950–2022.

## Data Availability

Code used for meta-analysis is publicly available at: https://github.com/shk313/diagnostic-test-metaanalysis/tree/main/Leptospirosis. Data included in analyses can be found in Table S2.

## Abbreviations

CrI: Credible interval
DPO: Days post-onset
FIEBRE: Febrile Illness Evaluation in a Broad Range of Endemicities
MAT: Microscopic agglutination test
PCR: Polymerase chain reaction
PROSPERO: International Prospective Register of Systematic Reviews
QUADAS-2: Quality Assessment of Studies of Diagnostic Accuracy Approach- 2
SROC: Summary Receiver Operating Characteristic
WHO: World Health Organization
