# Supplementary material for: Diagnosis of human leptospirosis: systematic review and meta-analysis of the diagnostic accuracy of the Leptospira microscopic agglutination test, PCR targeting Lfb1, and IgM ELISA to Leptospira fainei serovar Hurstbridge
Source: BMC Infect Dis. 2024 Feb 7;24:168. doi: 10.1186/s12879-023-08935-0 (PMC10848445; doi:10.1186/s12879-023-08935-0)
Supplement: Supplementary file 1 — Additional file 1: Appendix S1. Search strategy for the systematic review of studies evaluating the diagnostic accuracy of MAT, PCR, and IgM ELISA, published global and between 1950–2022. [file 12879_2023_8935_MOESM1_ESM.docx]

Appendix S1: Search strategy for the systematic review of studies evaluating the diagnostic accuracy of MAT, PCR, and IgM ELISA, published global and between 1950 –2022.

Table of Contents

[1 Search methodology 2](#_Toc120884946)

[2 Databases 3](#_Toc120884947)

[3 Information management 3](#_Toc120884948)

[4 Results 4](#_Toc120884949)

[4.1 PCR test search 4](#_Toc120884950)

[4.2 Agglutination test search 4](#_Toc120884951)

[4.3 ELISA test search 5](#_Toc120884952)

[5 References 6](#_Toc120884953)

[6 Appendix: Search strategies 6](#_Toc120884954)

[6.1 PCR test searches 6](#_Toc120884955)

[6.1.1 OvidSP Medline 6](#_Toc120884956)

[6.1.2 OvidSP Embase 8](#_Toc120884957)

[6.1.3 OvidSP Global Health 10](#_Toc120884958)

[6.1.4 Wiley Cochrane CENTRAL database 11](#_Toc120884959)

[6.1.5 Clarivate Analytics Web of Science 13](#_Toc120884960)

[6.1.6 Ebsco Africa-Wide Information 14](#_Toc120884961)

[6.1.7 Scopus 16](#_Toc120884962)

[6.1.8 LILACS 17](#_Toc120884963)

[6.1.9 Global Index Medicus 18](#_Toc120884964)

[6.2 Agglutination test searches 18](#_Toc120884965)

[6.2.1 OvidSP Medline 18](#_Toc120884966)

[6.2.2 OvidSP Embase 20](#_Toc120884967)

[6.2.3 OvidSP Global Health 21](#_Toc120884968)

[6.2.4 Wiley Cochrane CENTRAL database 23](#_Toc120884969)

[6.2.5 Clarivate Analytics Web of Science 24](#_Toc120884970)

[6.2.6 Ebsco Africa-Wide Information 25](#_Toc120884971)

[6.2.7 Scopus 26](#_Toc120884972)

[6.2.8 LILACS 27](#_Toc120884973)

[6.2.9 Global Index Medicus 28](#_Toc120884974)

[6.3 ELISA test searches 29](#_Toc120884975)

[6.3.1 OvidSP Medline 29](#_Toc120884976)

[6.3.2 OvidSP Embase 30](#_Toc120884977)

[6.3.3 OvidSP Global Health 32](#_Toc120884978)

[6.3.4 Wiley Cochrane CENTRAL database 33](#_Toc120884979)

[6.3.5 Clarivate Analytics Web of Science 35](#_Toc120884980)

[6.3.6 Ebsco Africa-Wide Information 36](#_Toc120884981)

[6.3.7 Scopus 38](#_Toc120884982)

[6.3.8 LILACS 39](#_Toc120884983)

[6.3.9 Global Index Medicus 39](#_Toc120884984)

# Search methodology

Three draft search strategies were compiled in the OvidSP Medline database by an experienced information specialist (JF). The search strategies included strings of terms, synonyms and controlled vocabulary terms (where available) to reflect two concepts:

Concept 1: leptospirosis

Concept 2: diagnostic test:

1. PCR test
2. Agglutination test
3. ELISA test

As recommended in the Cochrane Handbook for Systematic Reviews of Diagnostic Test Accuracy, a specific filter to identify diagnostic test accuracy studies was not included.^1^ Animal studies were excluded using the relevant section of the Cochrane RCT filter.^2^ The PCR and agglutination test searches were limited by date from 1950 to present. The ELISA search had no limitations by date. No other limits were applied. This search strategies were refined with the project team until the results retrieved reflected the scope of the project. The agreed OvidSP Medline searches were adapted for each database to incorporate database-specific syntax and controlled vocabularies. Full details of the search strings used for each database can be found in the appendix.

# Databases

Searches for all three tests were conducted on 16 August 2022 on the following databases:

- OvidSP Medline ALL, 1946 to August 15, 2022.
- OvidSP Embase Classic+Embase, 1947 to 2022 August 15
- OvidSP Global Health, 1910 to 2022 week 32.
- Wiley Cochrane Central Register of Controlled Trials, Issue 08 of 12, August 2022.
- Clarivate Analytics Web of Science:
  - Science Citation Index-Expanded, 1970-present;
  - Social Sciences Citation Index, 1970-present.
- Elsevier Scopus, complete database.
- Ebsco Africa-Wide Information, complete database.
- WHO LILACS, complete database.
- WHO Global Index Medicus, complete database.

# Information management

All citations identified by our searches were imported into EndNote 20 software, with results from each test search imported into a different EndNote library. Duplicates were identified and removed using the method described on the London School of Hygiene & Tropical Medicine Library & Archives Service blog.^3^

# Results

## PCR test search

A total of 2398 results were retrieved by the search. 1310 (55%) were identified as duplicates. Number of results pre-and post-deduplication are listed in the table below.

| Database name | Total number of results | Number of results once duplicates removed |
| --- | --- | --- |
| Medline ALL | 430 | 430 |
| Embase Classic+Embase | 561 | 293 |
| Global Health | 298 | 102 |
| Africa-Wide Information | 22 | 5 |
| Cochrane Central Register of Controlled Trials | 2 | 1 |
| Global Index Medicus | 23 | 17 |
| Web of Science databases (both searched together) | 400 | 96 |
| Scopus | 658 | 140 |
| LILACS | 4 | 4 |
| **Total** | **2398** | **1088** |

## Agglutination test search

A total of 1453 results were retrieved by the search. 787 (54%) were identified as duplicates. Number of results pre-and post-deduplication are listed in the table below.

| Database name | Total number of results | Number of results once duplicates removed |
| --- | --- | --- |
| Medline ALL | 193 | 192 |
| Embase Classic+Embase | 306 | 173 |
| Global Health | 217 | 110 |
| Africa-Wide Information | 21 | 0 |
| Cochrane Central Register of Controlled Trials | 1 | 0 |
| Global Index Medicus | 69 | 8 |
| Web of Science databases (both searched together) | 207 | 44 |
| Scopus | 402 | 124 |
| LILACS | 37 | 15 |
| **Total** | **1453** | **666** |

## ELISA test search

A total of 10,788 results were retrieved by the search. 5701 (53%) were identified as duplicates. Number of results pre-and post-deduplication are listed in the table below.

| Database name | Total number of results | Number of results once duplicates removed |
| --- | --- | --- |
| Medline ALL | 1465 | 1443 |
| Embase Classic+Embase | 3023 | 1922 |
| Global Health | 1725 | 816 |
| Africa-Wide Information | 198 | 16 |
| Cochrane Central Register of Controlled Trials | 15 | 3 |
| Global Index Medicus | 656 | 93 |
| Web of Science databases (both searched together) | 1411 | 286 |
| Scopus | 1888 | 232 |
| LILACS | 407 | 276 |
| **Total** | **10,788** | **5087** |

# References

1. de Vet HCW, Eisinga A, Riphagen II, Aertgeerts B, Pewsner D. Searching for Studies. Cochrane Handbook for Systematic Reviews of Diagnostic Test Accuracy: The Cochrane Collaboration; 2008. Available from: <https://methods.cochrane.org/sites/methods.cochrane.org.sdt/files/public/uploads/Chapter07-Searching-%28September-2008%29.pdf>

2. Lefebvre C, Glanville J, Briscoe S, Littlewood A, Marshall C, Metzendorf MI, et al. Technical Supplement to Chapter 4: Searching for and selecting studies. In: Higgins JPT, Thomas J, Chandler J, Cumpston MS, Li T, Page MJ, et al., editors. Cochrane Handbook for Systematic Reviews of Interventions 6th ed: Cochrane; 2019. Available from: <https://training.cochrane.org/handbook/version-6/chapter-4-tech-suppl>.

3. Falconer J. Removing duplicates from an EndNote Library. Library & Archives Service Blog [Internet]: London School of Hygiene & Tropical Medicine. 2018. [cited 2020]. Available from: <https://blogs.lshtm.ac.uk/library/2018/12/07/removing-duplicates-from-an-endnote-library/>.

# Appendix: Search strategies

This appendix provides full details of all search strings used for bibliographic databases, with dates and number of references returned and notes explaining any unusual search techniques or syntax. The EndNote 20 import order is provided, as the deduplication technique keeps the first uploaded copy of the reference by default.

## PCR test searches

### OvidSP Medline

| Database name | Medline ALL |
| --- | --- |
| Database platform | OvidSP |
| Dates of database coverage | 1946 to August 15, 2022 |
| Date searched | 16 August 2022 |
| Searched by | JF |
| Number of results | 430 |
| EndNote import order | 1 |
| Number of results once duplicates removed | 430 |
| Search strategy notes | Search lines ending in a ‘/’ are subject heading searches. Search lines beginning ‘exp’ are exploded subject heading searches. Two-letter codes at the end of search lines designate the fields to search. Fields codes used are: TI: title AB: abstract KF: author keywords SH: subject heading adj*n* searches for words within *n* words of each other. or/*x-y* combines search sets in the range *x-y* with Boolean operator OR. * is used for truncation of words. |

| **#** | **Search terms** | **Results** |
| --- | --- | --- |
| 1 | (lfb1 or lfb-1).ti,ab,kf. | 49 |
| 2 | (leptospir* adj3 (dna or gene*)).ti,ab,kf. | 699 |
| 3 | exp Leptospirosis/ | 9145 |
| 4 | exp Leptospiraceae/ | 6683 |
| 5 | leptospir*.ti,ab,kf. | 12877 |
| 6 | or/3-5 | 13816 |
| 7 | DNA, Bacterial/ | 118051 |
| 8 | 6 and 7 | 602 |
| 9 | 1 or 2 or 8 | 1132 |
| 10 | Molecular Diagnostic Techniques/ | 13519 |
| 11 | ((molecular or DNA or nucleic acid) adj5 (assay* or amplif* or detect* or diagnos* or technique* or test*)).ti,ab,kf. | 270857 |
| 12 | Nucleic Acid Amplification Techniques/ | 13186 |
| 13 | exp Polymerase Chain Reaction/ | 463732 |
| 14 | (polymerase chain reaction* or PCR* or qPCR* or rtPCR* or NAAT or NASBA or self-sustained sequence replicat* or isothermal amplif* or LAMP or primer or primers).ti,ab,kf. | 835970 |
| 15 | or/10-14 | 1200845 |
| 16 | 9 and 15 | 674 |
| 17 | exp animals/ not humans.sh. | 5037247 |
| 18 | 16 not 17 | 433 |
| 19 | limit 18 to yr="1950 -Current" | 433 |
| 20 | remove duplicates from 19 | 430 |

### OvidSP Embase

| Database name | Embase Classic+Embase |
| --- | --- |
| Database platform | OvidSP |
| Dates of database coverage | 1947 to 2022 August 15 |
| Date searched | 16 August 2022 |
| Searched by | JF |
| Number of results | 561 |
| EndNote import order | 2 |
| Number of results once duplicates removed | 293 |
| Search strategy notes | Search lines ending in a ‘/’ are subject heading searches. Search lines beginning ‘exp’ are exploded subject heading searches. Two-letter codes at the end of search lines designate the fields to search. Fields codes used are: TI: title AB: abstract KW: author keywords SH: subject heading adj*n* searches for words within *n* words of each other. or/*x-y* combines search sets in the range *x-y* with Boolean operator OR. * is used for truncation of words. |

| **#** | **Search terms** | **Results** |
| --- | --- | --- |
| 1 | (lfb1 or lfb-1).ti,ab,kw. | 55 |
| 2 | (leptospir* adj3 (dna or gene*)).ti,ab,kw. | 803 |
| 3 | leptospirosis/ | 12962 |
| 4 | exp leptospiraceae/ | 9078 |
| 5 | leptospir*.ti,ab,kw. | 15671 |
| 6 | or/3-5 | 18304 |
| 7 | bacterial DNA/ | 61887 |
| 8 | 6 and 7 | 397 |
| 9 | 1 or 2 or 8 | 1079 |
| 10 | molecular diagnosis/ | 25692 |
| 11 | ((molecular or DNA or nucleic acid) adj5 (assay* or amplif* or detect* or diagnos* or technique* or test*)).ti,ab,kw. | 340850 |
| 12 | nucleic acid amplification/ | 10929 |
| 13 | exp polymerase chain reaction/ | 1114981 |
| 14 | (polymerase chain reaction* or PCR* or qPCR* or rtPCR* or NAAT or NASBA or self-sustained sequence replicat* or isothermal amplif* or LAMP or primer or primers).ti,ab,kw. | 1144759 |
| 15 | or/10-14 | 1726692 |
| 16 | 9 and 15 | 652 |
| 17 | (rat or rats or mouse or mice or swine or porcine or murine or sheep or lambs or pigs or piglets or rabbit or rabbits or cat or cats or dog or dogs or cattle or bovine or monkey or monkeys or trout or marmoset$1).ti. and animal experiment/ | 1161698 |
| 18 | Animal experiment/ not (human experiment/ or human/) | 2441371 |
| 19 | 17 or 18 | 2502392 |
| 20 | 16 not 19 | 565 |
| 21 | limit 20 to yr="1950 -Current" | 564 |
| 22 | remove duplicates from 21 | 561 |

### OvidSP Global Health

| Database name | Global Health |
| --- | --- |
| Database platform | OvidSP |
| Dates of database coverage | 1910 to 2022 week 32 |
| Date searched | 16 August 2022 |
| Searched by | JF |
| Number of results | 298 |
| EndNote import order | 3 |
| Number of results once duplicates removed | 102 |
| Search strategy notes | Search lines ending in a ‘/’ are subject heading searches. Search lines beginning ‘exp’ are exploded subject heading searches. Two-letter codes at the end of search lines designate the fields to search. Fields codes used are: TI: title AB: abstract SH: subject heading adj*n* searches for words within *n* words of each other. or/*x-y* combines search sets in the range *x-y* with Boolean operator OR. * is used for truncation of words. |

| **#** | **Search terms** | **Results** |
| --- | --- | --- |
| 1 | (lfb1 or lfb-1).ti,ab. | 10 |
| 2 | (leptospir* adj3 (dna or gene*)).ti,ab. | 476 |
| 3 | exp leptospiraceae/ | 10688 |
| 4 | leptospir*.ti,ab. | 10178 |
| 5 | 3 or 4 | 11153 |
| 6 | dna/ or dna probes/ | 51520 |
| 7 | 5 and 6 | 222 |
| 8 | 1 or 2 or 7 | 580 |
| 9 | ((molecular or DNA or nucleic acid) adj5 (assay* or amplif* or detect* or diagnos* or technique* or test*)).ti,ab. | 67090 |
| 10 | exp polymerase chain reaction/ | 68093 |
| 11 | (polymerase chain reaction* or PCR* or qPCR* or rtPCR* or NAAT or NASBA or self-sustained sequence replicat* or isothermal amplif* or LAMP or primer or primers).ti,ab. | 216343 |
| 12 | or/9-11 | 249416 |
| 13 | 8 and 12 | 391 |
| 14 | ((rat or rats or mouse or mice or swine or porcine or murine or sheep or lambs or pigs or piglets or rabbit or rabbits or cat or cats or dog or dogs or cattle or bovine or monkey or monkeys or trout or marmoset$1) not man).sh. | 549844 |
| 15 | 13 not 14 | 298 |
| 16 | limit 15 to yr="1950 -Current" | 298 |
| 17 | remove duplicates from 16 | 298 |

### Wiley Cochrane CENTRAL database

| Database name | Cochrane Central Register of Controlled Trials |
| --- | --- |
| Database platform | Wiley |
| Dates of database coverage | Issue 8 of 12, August 2022 |
| Date searched | 16 August 2022 |
| Searched by | JF |
| Number of results | 2 |
| EndNote import order | 7 |
| Number of results once duplicates removed | 1 |
| Search strategy notes | * is used for truncation.  NEAR/n finds terms within n words of each other in any order.  NEXT finds terms next to each other in the order they have been entered. This is used for phrase searching where truncation is used.  # is used for compulsory wildcards  ? is used for optional wildcards  Searches ending :ti,ab,kw search the title, abstract and keywords.  Note, results are number of items retrieved across all Cochrane databases. |

| **#** | **Search terms** | **Results** |
| --- | --- | --- |
| #1 | (lfb1 or "lfb-1"):ti,ab,kw | 1 |
| #2 | (leptospir* NEAR/3 (dna or gene*)):ti,ab,kw | 3 |
| #3 | MeSH descriptor: [Leptospirosis] explode all trees | 36 |
| #4 | MeSH descriptor: [Leptospiraceae] explode all trees | 11 |
| #5 | (leptospir*):ti,ab,kw | 92 |
| #6 | #3 OR #4 OR #5 | 92 |
| #7 | MeSH descriptor: [DNA, Bacterial] this term only | 276 |
| #8 | #6 AND #7 | 0 |
| #9 | #1 OR #2 OR #8 | 4 |
| #10 | MeSH descriptor: [Molecular Diagnostic Techniques] this term only | 51 |
| #11 | ((molecular or DNA or "nucleic acid") NEAR/5 (assay* or amplif* or detect* or diagnos* or technique* or test*)):ti,ab,kw | 5036 |
| #12 | MeSH descriptor: [Nucleic Acid Amplification Techniques] this term only | 40 |
| #13 | MeSH descriptor: [Polymerase Chain Reaction] explode all trees | 2156 |
| #14 | (("polymerase chain" NEXT reaction*) or PCR* or qPCR* or rtPCR* or NAAT or NASBA or ("self-sustained sequence" NEXT replicat*) or (isothermal NEXT amplif*) or LAMP or primer or primers):ti,ab,kw | 24941 |
| #15 | #10 OR #11 OR #12 OR #13 OR #14 | 28388 |
| #16 | #9 AND #15 | 2 |

### Clarivate Analytics Web of Science

| Database name | Science Citation Index Expanded Social Sciences Citation Index |
| --- | --- |
| Database platform | Clarivate Analytics Web of Science |
| Dates of database coverage | Both databases 1970-present. |
| Date searched | 16 August 2022 |
| Searched by | JF |
| Number of results | 400 |
| EndNote import order | 5 |
| Number of results once duplicates removed | 96 |
| Search strategy notes | * is used for truncation.  TOPIC searches search in the title, abstract and keywords fields.  NEAR/*n* searches for words within *n* words of each other.  All searches run across Indexes=SCI-EXPANDED, SSCI. Timespan=All years |

| **#** | **Search terms** | **Results** |
| --- | --- | --- |
| 1 | TS=(lfb1 or "lfb-1") | 79 |
| 2 | TS=(leptospir* NEAR/3 (dna or gene*) ) | 747 |
| 3 | #2 OR #1 | 821 |
| 4 | TS=((molecular or DNA or "nucleic acid") NEAR/5 (assay* or amplif* or detect* or diagnos* or technique* or test*) ) | 323752 |
| 5 | TS=("polymerase chain reaction*" or PCR* or qPCR* or rtPCR* or NAAT or NASBA or "self-sustained sequence replicat*" or "isothermal amplif*" or LAMP or primer or primers) | 929933 |
| 6 | #5 OR #4 | 1141036 |
| 7 | #6 AND #3 | 450 |
| 8 | TI=(rat or rats or mouse or mice or swine or porcine or murine or sheep or lambs or pigs or piglets or rabbit or rabbits or cat or cats or dog or dogs or cattle or bovine or monkey or monkeys or trout or marmoset*) NOT TS=(man or human) | 2383353 |
| 9 | #7 NOT #8 | 400 |

### Ebsco Africa-Wide Information

| Database name | Africa-Wide Information |
| --- | --- |
| Database platform | Ebsco |
| Dates of database coverage | Complete database as of search date |
| Date searched | 16 August 2022 |
| Searched by | JF |
| Number of results | 22 |
| EndNote import order | 4 |
| Number of results once duplicates removed | 5 |
| Search strategy notes | Two-letter codes at the beginning of search lines designate the fields to search. Fields codes used are: TI: title AB: abstract KW: keywords * is used for truncation of words. N*n* searches for terms within *n* words of each other. |

| **#** | **Search terms** | **Results** |
| --- | --- | --- |
| S1 | (TI (lfb1 OR lfb-1)) OR (AB (lfb1 OR lfb-1)) OR (KW (lfb1 OR lfb-1)) | 1 |
| S2 | (TI (leptospir* N3 (dna or gene*)) OR (AB (leptospir* N3 (dna or gene*)) OR (KW (leptospir* N3 (dna or gene*)) | 48 |
| S3 | (TI ((molecular or DNA or "nucleic acid") N5 (assay* or amplif* or detect* or diagnos* or technique* or test*))) OR (AB ((molecular or DNA or "nucleic acid") N5 (assay* or amplif* or detect* or diagnos* or technique* or test*))) OR (KW ((molecular or DNA or "nucleic acid") N5 (assay* or amplif* or detect* or diagnos* or technique* or test*))) | 20,423 |
| S4 | (TI ("polymerase chain reaction*" or PCR* or qPCR* or rtPCR* or NAAT or NASBA or "self-sustained sequence replicat*" or "isothermal amplif*" or LAMP or primer or primers)) OR (AB ("polymerase chain reaction*" or PCR* or qPCR* or rtPCR* or NAAT or NASBA or "self-sustained sequence replicat*" or "isothermal amplif*" or LAMP or primer or primers)) OR (KW ("polymerase chain reaction*" or PCR* or qPCR* or rtPCR* or NAAT or NASBA or "self-sustained sequence replicat*" or "isothermal amplif*" or LAMP or primer or primers)) | 51,729 |
| S5 | S1 OR S2 | 48 |
| S6 | S3 OR S4 | 62,104 |
| S7 | S5 AND S6 | 27 |
| S8 | (TI (rat or rats or mouse or mice or swine or porcine or murine or sheep or lambs or pigs or piglets or rabbit or rabbits or cat or cats or dog or dogs or cattle or bovine or monkey or monkeys or trout or marmoset*)) | 84,864 |
| S9 | (TI (man or human)) OR (AB (man or human)) OR (KW (man or human)) | 535,380 |
| S10 | S8 NOT S9 | 74,626 |
| S11 | S7 NOT S10 | 22 |
| S12 | S11 Limiters - Year Published: 1950-2023 | 22 |

### Scopus

| Database name | Scopus |
| --- | --- |
| Database platform | Scopus.com |
| Dates of database coverage | Complete database as of search date |
| Date searched | 16 August 2022 |
| Searched by | JF |
| Number of results | 658 |
| EndNote import order | 6 |
| Number of results once duplicates removed | 140 |
| Search strategy notes | * is used for truncation of words.  W/*n* finds terms within *n* words of each other.  { } searches for exact term with no lemmatization or stemming. |

| **Search terms** | **Results** |
| --- | --- |
| ( ( TITLE-ABS-KEY ( lfb1 OR "lfb-1" OR ( leptospir* W/3 ( "dna" OR gene* ) ) ) ) AND ( ( TITLE-ABS-KEY ( ( molecular OR dna OR "nucleic acid" ) W/5 ( assay* OR amplif* OR detect* OR diagnos* OR technique* OR test ) ) ) OR ( TITLE-ABS-KEY ( "polymerase chain reaction*" OR pcr* OR qpcr* OR rtpcr* OR naat OR nasba OR "self-sustained sequence replicat*" OR "isothermal amplif*" OR {LAMP} OR {primer} OR {primers} ) ) ) ) AND NOT ( ( ( TITLE ( {rat} OR {rats} OR {mouse} OR {mice} OR {swine} OR {porcine} OR {murine} OR {sheep} OR {lambs} OR {pigs} OR {piglets} OR {rabbit} OR {rabbits} OR {cat} OR {cats} OR {dog} OR {dogs} OR {cattle} OR {bovine} OR {monkey} OR {monkeys} OR {trout} OR marmoset* ) ) ) AND NOT ( ( TITLE-ABS-KEY ( {man} OR {human} ) ) ) ) | 658 |

### LILACS

| Database name | LILACS |
| --- | --- |
| Database platform | WHO Global Index Medicus |
| Dates of database coverage | Complete database as of search date |
| Date searched | 16 August 2022 |
| Searched by | JF |
| Number of results | 4 |
| EndNote import order | 8 |
| Number of results once duplicates removed | 4 |
| Search strategy notes | * is used for truncation of words. |

| **Search terms** | **Results** |
| --- | --- |
| (tw:(lfb1 OR "lfb-1" OR (leptosir* AND (dna OR gene*)))) AND (tw:(molecular OR dna OR "nucleic acid" OR "polymerase chain" OR PCR* OR qPCR* OR rtPCR* OR NAAT OR NASBA OR "self-sustained sequence" OR "isothermal amplification" OR LAMP OR primer OR primers)) | 4 |

### Global Index Medicus

| Database name | Global Index Medicus |
| --- | --- |
| Database platform | WHO Global Index Medicus |
| Dates of database coverage | Complete database as of search date |
| Date searched | 16 August 2022 |
| Searched by | JF |
| Number of results | 23 |
| EndNote import order | 9 |
| Number of results once duplicates removed | 17 |
| Search strategy notes | * is used for truncation of words. |

| **Search terms** | **Results** |
| --- | --- |
| (tw:(lfb1 OR "lfb-1" OR (leptosir* AND (dna OR gene*)))) AND (tw:(molecular OR dna OR "nucleic acid" OR "polymerase chain" OR PCR* OR qPCR* OR rtPCR* OR NAAT OR NASBA OR "self-sustained sequence" OR "isothermal amplification" OR LAMP OR primer OR primers)) | 23 |

## Agglutination test searches

### OvidSP Medline

| Database name | Medline ALL |
| --- | --- |
| Database platform | OvidSP |
| Dates of database coverage | 1946 to August 15, 2022 |
| Date searched | 16 August 2022 |
| Searched by | JF |
| Number of results | 193 |
| EndNote import order | 1 |
| Number of results once duplicates removed | 192 |
| Search strategy notes | Search lines ending in a ‘/’ are subject heading searches. Search lines beginning ‘exp’ are exploded subject heading searches. Two-letter codes at the end of search lines designate the fields to search. Fields codes used are: TI: title AB: abstract KF: author keywords SH: subject heading adj*n* searches for words within *n* words of each other. or/*x-y* combines search sets in the range *x-y* with Boolean operator OR. * is used for truncation of words. |

| **#** | **Search terms** | **Results** |
| --- | --- | --- |
| 1 | exp Leptospirosis/ | 9145 |
| 2 | exp Leptospiraceae/ | 6683 |
| 3 | leptospir*.ti,ab,kf. | 12877 |
| 4 | or/1-3 | 13816 |
| 5 | exp agglutination tests/ | 39780 |
| 6 | (agglutination or microagglutination or MAT or bMAT).ti,ab,kf. | 33357 |
| 7 | 5 or 6 | 64408 |
| 8 | exp Culture Techniques/ | 233457 |
| 9 | exp cell culture techniques/ | 67589 |
| 10 | culture*.ti,ab,kf. | 1125876 |
| 11 | or/8-10 | 1240933 |
| 12 | 4 and 7 and 11 | 313 |
| 13 | exp animals/ not humans.sh. | 5037247 |
| 14 | 12 not 13 | 195 |
| 15 | limit 14 to yr="1950 -Current" | 193 |
| 16 | remove duplicates from 15 | 193 |

### OvidSP Embase

| Database name | Embase Classic+Embase |
| --- | --- |
| Database platform | OvidSP |
| Dates of database coverage | 1947 to 2022 August 15 |
| Date searched | 16 August 2022 |
| Searched by | JF |
| Number of results | 306 |
| EndNote import order | 2 |
| Number of results once duplicates removed | 173 |
| Search strategy notes | Search lines ending in a ‘/’ are subject heading searches. Search lines beginning ‘exp’ are exploded subject heading searches. Two-letter codes at the end of search lines designate the fields to search. Fields codes used are: TI: title AB: abstract KW: author keywords SH: subject heading adj*n* searches for words within *n* words of each other. or/*x-y* combines search sets in the range *x-y* with Boolean operator OR. * is used for truncation of words. |

| **#** | **Search terms** | **Results** |
| --- | --- | --- |
| 1 | leptospirosis/ | 12962 |
| 2 | exp leptospiraceae/ | 9078 |
| 3 | leptospir*.ti,ab,kw. | 15671 |
| 4 | or/1-3 | 18304 |
| 5 | agglutination test/ | 16524 |
| 6 | (microscopic agglutination test* or microagglutination test* or MAT or bMAT).ti,ab,kw. | 15097 |
| 7 | 5 or 6 | 30136 |
| 8 | exp cell culture/ | 759454 |
| 9 | culture*.ti,ab,kw. | 1457655 |
| 10 | 8 or 9 | 1802352 |
| 11 | 4 and 7 and 10 | 347 |
| 12 | (rat or rats or mouse or mice or swine or porcine or murine or sheep or lambs or pigs or piglets or rabbit or rabbits or cat or cats or dog or dogs or cattle or bovine or monkey or monkeys or trout or marmoset$1).ti. and animal experiment/ | 1161698 |
| 13 | Animal experiment/ not (human experiment/ or human/) | 2441371 |
| 14 | 12 or 13 | 2502392 |
| 15 | 11 not 14 | 309 |
| 16 | limit 15 to yr="1950 -Current" | 308 |
| 17 | remove duplicates from 16 | 306 |

### OvidSP Global Health

| Database name | Global Health |
| --- | --- |
| Database platform | OvidSP |
| Dates of database coverage | 1910 to 2022 week 32 |
| Date searched | 16 August 2022 |
| Searched by | JF |
| Number of results | 217 |
| EndNote import order | 3 |
| Number of results once duplicates removed | 110 |
| Search strategy notes | Search lines ending in a ‘/’ are subject heading searches. Search lines beginning ‘exp’ are exploded subject heading searches. Two-letter codes at the end of search lines designate the fields to search. Fields codes used are: TI: title AB: abstract SH: subject heading adj*n* searches for words within *n* words of each other. or/*x-y* combines search sets in the range *x-y* with Boolean operator OR. * is used for truncation of words. |

| **#** | **Search terms** | **Results** |
| --- | --- | --- |
| 1 | exp leptospira/ | 10686 |
| 2 | leptospir*.ti,ab. | 10178 |
| 3 | 1 or 2 | 11152 |
| 4 | exp agglutination tests/ | 7348 |
| 5 | (microscopic agglutination test* or microagglutination test* or MAT or bMAT).ti,ab. | 3741 |
| 6 | 4 or 5 | 9996 |
| 7 | exp culture techniques/ | 32773 |
| 8 | cell cultures/ | 28354 |
| 9 | culture*.ti,ab. | 268662 |
| 10 | or/7-9 | 291525 |
| 11 | 3 and 6 and 10 | 341 |
| 12 | ((rat or rats or mouse or mice or swine or porcine or murine or sheep or lambs or pigs or piglets or rabbit or rabbits or cat or cats or dog or dogs or cattle or bovine or monkey or monkeys or trout or marmoset$1) not man).sh. | 549844 |
| 13 | 11 not 12 | 248 |
| 14 | limit 13 to yr="1950 -Current" | 219 |
| 15 | remove duplicates from 14 | 217 |

### Wiley Cochrane CENTRAL database

| Database name | Cochrane Central Register of Controlled Trials |
| --- | --- |
| Database platform | Wiley |
| Dates of database coverage | Issue 8 of 12, August 2022 |
| Date searched | 16 August 2022 |
| Searched by | JF |
| Number of results | 1 |
| EndNote import order | 7 |
| Number of results once duplicates removed | 0 |
| Search strategy notes | * is used for truncation.  NEAR/n finds terms within n words of each other in any order.  NEXT finds terms next to each other in the order they have been entered. This is used for phrase searching where truncation is used.  # is used for compulsory wildcards  ? is used for optional wildcards  Searches ending :ti,ab,kw search the title, abstract and keywords.  Note, results are numbers retrieved across all Cochrane databases. |

| **#** | **Search terms** | **Results** |
| --- | --- | --- |
| #1 | MeSH descriptor: [Leptospirosis] explode all trees | 36 |
| #2 | MeSH descriptor: [Leptospiraceae] explode all trees | 11 |
| #3 | (leptospir*):ti,ab,kw | 92 |
| #4 | #1 OR #2 OR #3 | 92 |
| #5 | MeSH descriptor: [Agglutination Tests] explode all trees | 147 |
| #6 | (agglutination or microagglutination or MAT or bMAT):ti,ab,kw | 1298 |
| #7 | #5 OR #6 | 1384 |
| #8 | MeSH descriptor: [Culture Techniques] explode all trees | 549 |
| #9 | MeSH descriptor: [Cell Culture Techniques] explode all trees | 134 |
| #10 | (culture*):ti,ab,kw | 23501 |
| #11 | #8 OR #9 OR #10 | 23554 |
| #12 | #4 AND #7 AND #11 | 2 |

### Clarivate Analytics Web of Science

| Database name | Science Citation Index Expanded Social Sciences Citation Index |
| --- | --- |
| Database platform | Clarivate Analytics Web of Science |
| Dates of database coverage | Both databases 1970-present. |
| Date searched | 16 August 2022 |
| Searched by | JF |
| Number of results | 207 |
| EndNote import order | 5 |
| Number of results once duplicates removed | 44 |
| Search strategy notes | * is used for truncation.  TOPIC searches search in the title, abstract and keywords fields.  NEAR/*n* searches for words within *n* words of each other.  All searches run across Indexes=SCI-EXPANDED, SSCI. Timespan=All years |

| **#** | **Search terms** | **Results** |
| --- | --- | --- |
| 1 | TS=(leptospir*) | 10375 |
| 2 | TS=(agglutination or microagglutination or MAT or bMAT) | 54328 |
| 3 | TS=(culture*) | 1351705 |
| 4 | #3 AND #2 AND #1 | 264 |
| 5 | TI=(rat or rats or mouse or mice or swine or porcine or murine or sheep or lambs or pigs or piglets or rabbit or rabbits or cat or cats or dog or dogs or cattle or bovine or monkey or monkeys or trout or marmoset*) NOT TS=(man or human) | 2383353 |
| 6 | #4 NOT #5 | 207 |

### Ebsco Africa-Wide Information

| Database name | Africa-Wide Information |
| --- | --- |
| Database platform | Ebsco |
| Dates of database coverage | Complete database as of search date |
| Date searched | 16 August 2022 |
| Searched by | JF |
| Number of results | 21 |
| EndNote import order | 4 |
| Number of results once duplicates removed | 0 |
| Search strategy notes | Two-letter codes at the beginning of search lines designate the fields to search. Fields codes used are: TI: title AB: abstract KW: keywords * is used for truncation of words. N*n* searches for terms within *n* words of each other. |

| **#** | **Search terms** | **Results** |
| --- | --- | --- |
| S1 | (TI leptospir*) OR (AB leptospir*) OR (KW leptospir*) | 1,132 |
| S2 | (TI (agglutination or microagglutination or MAT or bMAT)) OR (AB (agglutination or microagglutination or MAT or bMAT)) OR (KW (agglutination or microagglutination or MAT or bMAT)) | 3,102 |
| S3 | (TI culture*) OR (AB culture*) OR (KW culture*) | 151,485 |
| S4 | S1 AND S2 AND S3 | 25 |
| S5 | (TI (rat or rats or mouse or mice or swine or porcine or murine or sheep or lambs or pigs or piglets or rabbit or rabbits or cat or cats or dog or dogs or cattle or bovine or monkey or monkeys or trout or marmoset*)) | 84,864 |
| S6 | (TI (man or human)) OR (AB (man or human)) OR (KW (man or human)) | 535,380 |
| S7 | S5 NOT S6 | 74,626 |
| S8 | S4 NOT S7 | 23 |
| S9 | S8 Limiters - Year Published: 1950-2023 | 21 |

### Scopus

| Database name | Scopus |
| --- | --- |
| Database platform | Scopus.com |
| Dates of database coverage | Complete database as of search date |
| Date searched | 16 August 2022 |
| Searched by | JF |
| Number of results | 402 |
| EndNote import order | 6 |
| Number of results once duplicates removed | 124 |
| Search strategy notes | * is used for truncation of words.  W/*n* finds terms within *n* words of each other.  { } searches for exact term with no lemmatization or stemming. |

| **Search terms** | **Results** |
| --- | --- |
| ( ( TITLE-ABS-KEY ( leptospir* ) ) AND ( TITLE-ABS-KEY ( agglutination OR microagglutination OR mat OR bmat ) ) AND ( TITLE-ABS-KEY ( culture* ) ) ) AND NOT ( ( TITLE ( {rat} OR {rats} OR {mouse} OR {mice} OR {swine} OR {porcine} OR {murine} OR {sheep} OR {lambs} OR {pigs} OR {piglets} OR {rabbit} OR {rabbits} OR {cat} OR {cats} OR {dog} OR {dogs} OR {cattle} OR {bovine} OR {monkey} OR {monkeys} OR {trout} OR marmoset* ) ) AND NOT ( TITLE-ABS-KEY ( {man} OR {human} ) ) ) | 402 |

### LILACS

| Database name | LILACS |
| --- | --- |
| Database platform | WHO Global Index Medicus |
| Dates of database coverage | Complete database as of search date |
| Date searched | 16 August 2022 |
| Searched by | JF |
| Number of results | 37 |
| EndNote import order | 8 |
| Number of results once duplicates removed | 15 |
| Search strategy notes | * is used for truncation of words. |

| **Search terms** | **Results** |
| --- | --- |
| (tw:(leptospir*)) AND (tw:(agglutination OR microagglutination OR MAT OR bMAT)) AND (tw:(culture*)) | 37 |

### Global Index Medicus

| Database name | Global Index Medicus |
| --- | --- |
| Database platform | WHO Global Index Medicus |
| Dates of database coverage | Complete database as of search date |
| Date searched | 16 August 2022 |
| Searched by | JF |
| Number of results | 69 |
| EndNote import order | 9 |
| Number of results once duplicates removed | 8 |
| Search strategy notes | * is used for truncation of words. |

| **Search terms** | **Results** |
| --- | --- |
| (tw:(leptospir*)) AND (tw:(agglutination OR microagglutination OR MAT OR bMAT)) AND (tw:(culture*)) | 69 |

## ELISA test searches

### OvidSP Medline

| Database name | Medline ALL |
| --- | --- |
| Database platform | OvidSP |
| Dates of database coverage | 1946 to August 15, 2022 |
| Date searched | 16 August 2022 |
| Searched by | JF |
| Number of results | 1465 |
| EndNote import order | 1 |
| Number of results once duplicates removed | 1443 |
| Search strategy notes | Search lines ending in a ‘/’ are subject heading searches. Search lines beginning ‘exp’ are exploded subject heading searches. Two-letter codes at the end of search lines designate the fields to search. Fields codes used are: TI: title AB: abstract KF: author keywords SH: subject heading adj*n* searches for words within *n* words of each other. or/*x-y* combines search sets in the range *x-y* with Boolean operator OR. * is used for truncation of words. |

| **#** | **Search terms** | **Results** |
| --- | --- | --- |
| 1 | exp Leptospirosis/ | 9145 |
| 2 | exp Leptospiraceae/ | 6683 |
| 3 | leptospir*.ti,ab,kf. | 12877 |
| 4 | or/1-3 | 13816 |
| 5 | exp Enzyme-Linked Immunosorbent Assay/ | 154331 |
| 6 | (enzyme-linked immunosorbent assay? or enzyme-linked immunoassay? or elisa).ti,ab,kf. | 246885 |
| 7 | exp immunoglobulin m/ and exp Immunologic Tests/ | 13136 |
| 8 | ((immunoglobulin m or IgM or antibod*) adj5 (assay* or amplif* or detect* or diagnos* or technique* or test*)).ti,ab,kf. | 159621 |
| 9 | serologic tests/ | 21616 |
| 10 | (serologic* adj5 (assay* or amplif* or detect* or diagnos* or technique* or test*)).ti,ab,kf. | 39700 |
| 11 | hurstbridge.ti,ab,kf. | 14 |
| 12 | or/5-11 | 473191 |
| 13 | 4 and 12 | 2272 |
| 14 | exp animals/ not humans.sh. | 5037247 |
| 15 | 13 not 14 | 1466 |
| 16 | remove duplicates from 15 | 1465 |

### OvidSP Embase

| Database name | Embase Classic+Embase |
| --- | --- |
| Database platform | OvidSP |
| Dates of database coverage | 1947 to 2022 August 15 |
| Date searched | 16 August 2022 |
| Searched by | JF |
| Number of results | 3023 |
| EndNote import order | 2 |
| Number of results once duplicates removed | 1922 |
| Search strategy notes | Search lines ending in a ‘/’ are subject heading searches. Search lines beginning ‘exp’ are exploded subject heading searches. Two-letter codes at the end of search lines designate the fields to search. Fields codes used are: TI: title AB: abstract KW: author keywords SH: subject heading adj*n* searches for words within *n* words of each other. or/*x-y* combines search sets in the range *x-y* with Boolean operator OR. * is used for truncation of words. |

| **#** | **Search terms** | **Results** |
| --- | --- | --- |
| 1 | leptospirosis/ | 12962 |
| 2 | exp leptospiraceae/ | 9078 |
| 3 | leptospir*.ti,ab,kw. | 15671 |
| 4 | or/1-3 | 18304 |
| 5 | exp enzyme linked immunosorbent assay/ | 438585 |
| 6 | (enzyme-linked immunosorbent assay? or enzyme-linked immunoassay? or elisa).ti,ab,kw. | 366486 |
| 7 | immunoglobulin M/ and exp immunological procedures/ | 38225 |
| 8 | ((immunoglobulin m or IgM or antibod*) adj5 (assay* or amplif* or detect* or diagnos* or technique* or test*)).ti,ab,kw. | 216458 |
| 9 | serodiagnosis/ | 50100 |
| 10 | (serologic* adj5 (assay* or amplif* or detect* or diagnos* or technique* or test*)).ti,ab,kw. | 55742 |
| 11 | hurstbridge.ti,ab,kw. | 15 |
| 12 | or/5-11 | 757997 |
| 13 | 4 and 12 | 3331 |
| 14 | (rat or rats or mouse or mice or swine or porcine or murine or sheep or lambs or pigs or piglets or rabbit or rabbits or cat or cats or dog or dogs or cattle or bovine or monkey or monkeys or trout or marmoset$1).ti. and animal experiment/ | 1161698 |
| 15 | Animal experiment/ not (human experiment/ or human/) | 2441371 |
| 16 | 14 or 15 | 2502392 |
| 17 | 13 not 16 | 3049 |
| 18 | remove duplicates from 17 | 3023 |

### OvidSP Global Health

| Database name | Global Health |
| --- | --- |
| Database platform | OvidSP |
| Dates of database coverage | 1910 to 2022 week 32 |
| Date searched | 16 August 2022 |
| Searched by | JF |
| Number of results | 1725 |
| EndNote import order | 3 |
| Number of results once duplicates removed | 816 |
| Search strategy notes | Search lines ending in a ‘/’ are subject heading searches. Search lines beginning ‘exp’ are exploded subject heading searches. Two-letter codes at the end of search lines designate the fields to search. Fields codes used are: TI: title AB: abstract SH: subject heading adj*n* searches for words within *n* words of each other. or/*x-y* combines search sets in the range *x-y* with Boolean operator OR. * is used for truncation of words. |

| **#** | **Search terms** | **Results** |
| --- | --- | --- |
| 1 | exp leptospira/ | 10686 |
| 2 | leptospir*.ti,ab. | 10178 |
| 3 | 1 or 2 | 11152 |
| 4 | elisa/ | 31084 |
| 5 | (enzyme-linked immunosorbent assay? or enzyme-linked immunoassay? or elisa).ti,ab. | 79961 |
| 6 | IgM/ and exp immunological techniques/ | 4968 |
| 7 | immunodiagnosis/ | 22142 |
| 8 | ((immunoglobulin m or IgM or antibod*) adj5 (assay* or amplif* or detect* or diagnos* or technique* or test*)).ti,ab. | 61722 |
| 9 | (serologic* adj5 (assay* or amplif* or detect* or diagnos* or technique* or test*)).ti,ab. | 25540 |
| 10 | hurstbridge.ti,ab. | 13 |
| 11 | or/4-10 | 148760 |
| 12 | 3 and 11 | 2367 |
| 13 | ((rat or rats or mouse or mice or swine or porcine or murine or sheep or lambs or pigs or piglets or rabbit or rabbits or cat or cats or dog or dogs or cattle or bovine or monkey or monkeys or trout or marmoset$1) not man).sh. | 549844 |
| 14 | 12 not 13 | 1733 |
| 15 | remove duplicates from 14 | 1725 |

### Wiley Cochrane CENTRAL database

| Database name | Cochrane Central Register of Controlled Trials |
| --- | --- |
| Database platform | Wiley |
| Dates of database coverage | Issue 8 of 12, August 2022 |
| Date searched | 16 August 2022 |
| Searched by | JF |
| Number of results | 15 |
| EndNote import order | 7 |
| Number of results once duplicates removed | 3 |
| Search strategy notes | * is used for truncation.  NEAR/n finds terms within n words of each other in any order.  NEXT finds terms next to each other in the order they have been entered. This is used for phrase searching where truncation is used.  # is used for compulsory wildcards  ? is used for optional wildcards  Searches ending :ti,ab,kw search the title, abstract and keywords.  Note, results are number of items retrieved across all Cochrane databases. |

| **#** | **Search Terms** | **Results** |
| --- | --- | --- |
| #1 | MeSH descriptor: [Leptospirosis] explode all trees | 36 |
| #2 | MeSH descriptor: [Leptospiraceae] explode all trees | 11 |
| #3 | (leptospir*):ti,ab,kw | 92 |
| #4 | #1 OR #2 OR #3 | 92 |
| #5 | MeSH descriptor: [Enzyme-Linked Immunosorbent Assay] explode all trees | 2498 |
| #6 | (("enzyme-linked immunosorbent" NEXT assay?) or ("enzyme-linked" NEXT immunoassay?) or elisa):ti,ab,kw | 15294 |
| #7 | MeSH descriptor: [Immunoglobulin M] explode all trees | 566 |
| #8 | MeSH descriptor: [Immunologic Tests] explode all trees | 5346 |
| #9 | (("immunoglobulin m" or IgM or antibod*) NEAR/5 (assay* or amplif* or detect* or diagnos* or technique* or test*)):ti,ab,kw | 4983 |
| #10 | MeSH descriptor: [Serologic Tests] this term only | 202 |
| #11 | (serologic* NEAR/5 (assay* or amplif* or detect* or diagnos* or technique* or test*)):ti,ab,kw | 966 |
| #12 | (hurstbridge):ti,ab,kw | 0 |
| #13 | #7 AND #8 | 79 |
| #14 | #5 OR #6 OR #13 OR #9 OR #10 OR #11 OR #12 | 19967 |
| #15 | #4 AND #14 | 17 |

### Clarivate Analytics Web of Science

| Database name | Science Citation Index Expanded Social Sciences Citation Index |
| --- | --- |
| Database platform | Clarivate Analytics Web of Science |
| Dates of database coverage | Both databases 1970-present. |
| Date searched | 16 August 2022 |
| Searched by | JF |
| Number of results | 1411 |
| EndNote import order | 5 |
| Number of results once duplicates removed | 286 |
| Search strategy notes | * is used for truncation.  TOPIC searches search in the title, abstract and keywords fields.  NEAR/*n* searches for words within *n* words of each other.  All searches run across Indexes=SCI-EXPANDED, SSCI. Timespan=All years |

| **#** | **Search terms** | **Results** |
| --- | --- | --- |
| 1 | TS=(leptospir*) | 10375 |
| 2 | TS=("enzyme-linked immunosorbent assay$" or "enzyme-linked immunoassay$" or elisa) | 232159 |
| 3 | TS=(("immunoglobulin m" or IgM or antibod*) NEAR/5 (assay* or amplif* or detect* or diagnos* or technique* or test*)) | 139263 |
| 4 | TS=(serologic* NEAR/5 (assay* or amplif* or detect* or diagnos* or technique* or test*)) | 32179 |
| 5 | TS=hurstbridge | 21 |
| 6 | #2 OR #3 OR #4 OR #5 | 353694 |
| 7 | TI=(rat or rats or mouse or mice or swine or porcine or murine or sheep or lambs or pigs or piglets or rabbit or rabbits or cat or cats or dog or dogs or cattle or bovine or monkey or monkeys or trout or marmoset*) NOT TS=(man or human) | 2383353 |
| 8 | #6 AND #1 | 1734 |
| 9 | #8 NOT #7 | 1411 |

### Ebsco Africa-Wide Information

| Database name | Africa-Wide Information |
| --- | --- |
| Database platform | Ebsco |
| Dates of database coverage | Complete database as of search date |
| Date searched | 16 August 2022 |
| Searched by | JF |
| Number of results | 198 |
| EndNote import order | 4 |
| Number of results once duplicates removed | 16 |
| Search strategy notes | Two-letter codes at the beginning of search lines designate the fields to search. Fields codes used are: TI: title AB: abstract KW: keywords * is used for truncation of words. N*n* searches for terms within *n* words of each other. |

| **#** | **Search terms** | **Results** |
| --- | --- | --- |
| S1 | (TI leptospir*) OR (AB leptospir*) OR (KW leptospir*) | 1,132 |
| S2 | (TI ("enzyme-linked immunosorbent assay*" OR "enzyme-linked immunoassay*" or elisa)) or (AB ("enzyme-linked immunosorbent assay*" OR "enzyme-linked immunoassay*" or elisa)) or (KW ("enzyme-linked immunosorbent assay*" OR "enzyme-linked immunoassay*" or elisa)) | 21,108 |
| S3 | (TI (("immunoglobulin m" or IgM or antibod*) N5 (assay* or amplif* or detect* or diagnos* or technique* or test*))) or (AB (("immunoglobulin m" or IgM or antibod*) N5 (assay* or amplif* or detect* or diagnos* or technique* or test*))) or (KW (("immunoglobulin m" or IgM or antibod*) N5 (assay* or amplif* or detect* or diagnos* or technique* or test*))) | 17,206 |
| S4 | (TI (serologic* N5 (assay* or amplif* or detect* or diagnos* or technique* or test*))) OR (AB (serologic* N5 (assay* or amplif* or detect* or diagnos* or technique* or test*))) or (KW (serologic* N5 (assay* or amplif* or detect* or diagnos* or technique* or test*))) | 6,003 |
| S5 | (TI hurstbridge) OR (AB hurstbridge) OR (KW hurstbridge) | 3 |
| S6 | S2 OR S3 OR S4 OR S5 | 35,602 |
| S7 | (TI (rat or rats or mouse or mice or swine or porcine or murine or sheep or lambs or pigs or piglets or rabbit or rabbits or cat or cats or dog or dogs or cattle or bovine or monkey or monkeys or trout or marmoset*)) | 84,864 |
| S8 | (TI (man or human)) OR (AB (man or human)) OR (KW (man or human)) | 535,380 |
| S9 | S7 not S8 | 74,626 |
| S10 | S1 AND S6 | 224 |
| S11 | s10 not s9 | 198 |

### Scopus

| Database name | Scopus |
| --- | --- |
| Database platform | Scopus.com |
| Dates of database coverage | Complete database as of search date |
| Date searched | 16 August 2022 |
| Searched by | JF |
| Number of results | 1888 |
| EndNote import order | 6 |
| Number of results once duplicates removed | 232 |
| Search strategy notes | * is used for truncation of words.  W/*n* finds terms within *n* words of each other.  { } searches for exact term with no lemmatization or stemming. |

| **Search terms** | **Results** |
| --- | --- |
| ( ( TITLE-ABS-KEY ( leptospir* ) ) AND ( TITLE-ABS-KEY ( “enzyme-linked immunosorbent assay*” OR “enzyme-linked immunoassay*” OR elisa OR hurstbridge OR ( (“immunoglobulin m” OR igm ) W/5 ( assay* OR mplify* OR detect* OR diagnos* OR technique* OR test* ) ) OR (serologic* W/5 ( assay* OR mplify* OR detect* OR diagnos* OR technique* OR test* ) ) ) ) ) AND NOT ( ( TITLE ( {rat} OR {rats} OR {mouse} OR {mice} OR {swine} OR {porcine} OR {murine} OR {sheep} OR {lambs} OR {pigs} OR {piglets} OR {rabbit} OR {rabbits} OR {cat} OR {cats} OR {dog} OR {dogs} OR {cattle} OR {bovine} OR {monkey} OR {monkeys} OR {trout} OR marmoset* ) ) AND NOT ( TITLE-ABS-KEY ( {man} OR {human} ) ) ) | 1888 |

### LILACS

| Database name | LILACS |
| --- | --- |
| Database platform | WHO Global Index Medicus |
| Dates of database coverage | Complete database as of search date |
| Date searched | 16 August 2022 |
| Searched by | JF |
| Number of results | 407 |
| EndNote import order | 8 |
| Number of results once duplicates removed | 276 |
| Search strategy notes | * is used for truncation of words. |

| **Search terms** | **Results** |
| --- | --- |
| (tw:(leptospir*)) AND (tw:("enzyme-linked immunosorbent assay" OR "enzyme-linked immunoassay" OR elisa OR "immunoglobulin m" OR IgM OR serologic* OR hurstbridge)) | 407 |

### Global Index Medicus

| Database name | Global Index Medicus |
| --- | --- |
| Database platform | WHO Global Index Medicus |
| Dates of database coverage | Complete database as of search date |
| Date searched | 16 August 2022 |
| Searched by | JF |
| Number of results | 656 |
| EndNote import order | 9 |
| Number of results once duplicates removed | 93 |
| Search strategy notes | * is used for truncation of words. |

| **Search terms** | **Results** |
| --- | --- |
| (tw:(leptospir*)) AND (tw:("enzyme-linked immunosorbent assay" OR "enzyme-linked immunoassay" OR elisa OR "immunoglobulin m" OR IgM OR serologic* OR hurstbridge)) | 656 |
