# Supplementary material for: Diagnosis of human leptospirosis: systematic review and meta-analysis of the diagnostic accuracy of the Leptospira microscopic agglutination test, PCR targeting Lfb1, and IgM ELISA to Leptospira fainei serovar Hurstbridge
Source: BMC Infect Dis. 2024 Feb 7;24:168. doi: 10.1186/s12879-023-08935-0 (PMC10848445; doi:10.1186/s12879-023-08935-0)
Supplement: Supplementary file 2 — Additional file 2: Appendix S2. Statistical model the systematic review of studies evaluating the diagnostic accuracy of MAT, PCR, and IgM ELISA, published global and between 1950–2022. Table S1. Sensitivity analysis in acute samples. Table S2. Sensitivity analysis in convalescent samples. [file 12879_2023_8935_MOESM2_ESM.docx]

**Appendix S2: Statistical model the systematic review of studies evaluating the diagnostic accuracy of MAT, PCR, and IgM ELISA, published global and between 1950 – 2022.**

The complete model to estimate the sensitivity and specificity of the index test in each study, and hence predict sensitivity and specificity in a future study, consists of two hierarchical levels. The first level captures between-study variations in the sensitivity and specificity through a Hierarchical Summary Receiver Operating Characteristic (HSROC) model (Rutter and Gatsonis, 2001; Dendukuri et al 2012; Dendukuri and Joseph, 2001), which assumes that sensitivity and specificity across studies lie on an ROC curve. The second level accounts for the unknown true disease status of all participants using latent class analysis.

In this analysis of the MAT diagnostic test, a separate meta-analysis was run for acute and convalescent samples. In the main text we present a model relaxing the assumption of conditional independence between test results from the same individual given their disease class. In the following, we first outline this model before presenting the results of a sensitivity analysis. We compared the result relaxing the assumption of conditional independence to those assuming conditional independence as well as presenting the results for a model where only studies from endemic settings are included. This is in comparison to the results presented in the main text where studies from both endemic and non-endemic settings are included.

Starting with the latent class model, assume we have a sample of N individuals who have all undergone two different dichotomous tests defined by $T_{r} (r =1,2), and let t_{ri}$ be a random variable denoting the outcome from test $r$for individual $i, i=1,..,N$. A positive test result for an individualis denoted by $t_{ri}=1$, and a negative test result by $t_{ri}=0.$ Also assume that the unknown true disease status of an individual, denoted $D$, can take one of two values: ‘diseased’ ($D=1)$ or ‘non-diseased’ ($D=0)$.The true (latent) disease status of the $i$^th^ individual is denoted $d_{i} .$ Assuming conditional independence between the two tests, we can write the joint distribution as:

| $\Pr\left( T_{1}= t_{1},T_{2}=t_{2} \right)=$  $Pr(T_{1}\vert D=1)Pr(T_{2}\vert D=1)Pr(D=1)+Pr(T_{1}\vert D=0)Pr(T_{2}\vert D=0)Pr(D=0)$ | 1 |
| --- | --- |

For the *r^t^*^h^ test, the sensitivity $(Se)$ and specificity $(Sp)$ can be written as $\mathrm{Se}_{r}=Pr(T_{r} =1|D=1)$ and$\mathrm{Sp}_{r}=Pr(T_{r}=0|D=0)$ respectively. We can also define prevalence as $\pi=\Pr(D=1)$. We can then specify equation 1 in terms of sensitivity, specificity and prevalence:

| $\Pr\left( T_{1}= t_{1},T_{2}=t_{2} \right)=$  $\mathrm{Se}_{1}\mathrm{Se}_{2}(1-\mathrm{Se}_{1})(1-\mathrm{Se}_{2})\pi+(1-\mathrm{Sp}_{1})(1-\mathrm{Sp}_{2})\mathrm{Sp}_{1}\mathrm{Sp}_{2}(1-\pi)$ | 2 |
| --- | --- |

Following this, we can then define the likelihood for this latent class model assuming conditional independence as:

| $L = \prod_{i=1}^{N} (\pi{\mathrm{Se}_{1}}^{t_{1i}}{\mathrm{Se}_{2}}^{t_{2i}}(1-{\mathrm{Se}_{1})}^{1-t_{1i}}(1-{\mathrm{Se}_{2})}^{1-t_{2i}})+((1-\pi){\mathrm{Sp}_{1}}^{1-t_{1i}}{\mathrm{Sp}_{2}}^{1-t_{2i}}(1-{\mathrm{Sp}_{1})}^{t_{1i}}(1-{\mathrm{Sp}_{2})}^{t_{2i}})$ | 3 |
| --- | --- |

To relax the assumption of conditional independence and account for conditional dependence between tests, we allow the sensitivity from each test to depend on an individual level random effect $s_{i}$. We assume that the sensitivity in the conditionally dependent model takes the form (1):

| ${Pr(T_{\mathrm{ri}}= 1\vert D_{i}=1,) =Se}_{\mathrm{ri}} = g^{-1}(a_{rd=1} + b_{rd=1}s_{i})$ | 4 |
| --- | --- |

where $g\left( \cdot\right)$ is a link function. In this study we use the logit link, $g^{-1}(y) = 1/(1+e^{-y})$, $a_{rd=1}$ and $b_{rd=1}$ are unknown parameters to be estimated where b describes the strength of dependence between two tests and the random effect $s_{i}$ follows a standard normal distribution. The subject-specific random-effect $s_{i}$ represents some unobserved characteristic for example infection intensity, that indirectly creates dependence between tests.

The study-level latent class model (equation 3 and 4) is then linked to the between-study level using a HSROC model (equation 5) which models between study variations by assuming that test sensitivity and specificity lie on an ROC curve. In particular, each study $j$ $(j=1,..,J)$provides the 2x2 table between the test of interest, hereafter called the index test, which is the same in all studies, and a comparator test which may differ between studies. We let $T_{1j}$ denote the index test outcomes in each study and we let $T_{2j}$ denote the comparator test outcomes in each study. In line with previous descriptions (2,3), we define the sensitivity and specificity of the index test in the $j$th study by:

| $\mathrm{logit}{(Se}_{1j})=Pr(T_{1j}=1\vert D=1) =-(\theta_{j} - \alpha_{j}/2)/exp(\beta/2)$  ${logit(Sp}_{1j})=Pr(T_{1j}=0\vert D=0) =(\theta_{j} + \alpha_{j}/2)/exp(-\beta/2)$ | 5 |
| --- | --- |

where $\theta_{j}$ represents the positivity criteria for study $j$. The positivity criteria, or cut-off value, models the dependence between the true positive fraction and false positive fraction in each study. $\alpha_{j}$ represents the diagnostic accuracy and measures the mean difference in test accuracy between individuals ‘diseased’ and individuals ‘non-diseased’ in study $j$. $\beta$ the scale parameter, allows differences in the variation of outcomes between disease positive and disease negative individuals thus allowing asymmetry in the ROC curve. Both $\theta$ and$\alpha$ parameters are modelled as random effects with independent normal distributions to incorporate variation between studies:

| $\theta_{j}\sim N(\Theta, \sigma_{\theta})$  $\alpha_{j} \sim N(\Lambda, \sigma_{\alpha})$ | 6 |
| --- | --- |

We present pooled estimates of sensitivity and specificity which are given by:

| $\mathrm{logit}\left( Pooled Se \right)=-\left( \left( \Theta-\Lambda/2 \right)/\exp\left( \beta/2 \right) \right)$  $logit (Pooled Sp)=((\Theta+\Lambda/2)/exp(-\beta/2))$ | 8 |
| --- | --- |

Importantly, we also present a prediction of sensitivity and specificity in a new study. Predicted estimates are important because the pooled estimates in a meta-analysis only represent an average estimate among the studies included in the analysis. Predicted estimates on the other hand account for the variation captured through the modelling framework and can be used as priors for sensitivity and specificity of the index test in a new study. We predict sensitivity and specificity by replacing $\Theta$ with $\theta_{newstudy}$ and $\Lambda$ with $\alpha_{newstudy}$:

| $\theta_{newstudy}\sim N\left( \Theta,\sigma_{\theta} \right)$  $\alpha_{newstudy}\sim N\left( \Lambda,\sigma_{\alpha} \right)$  The above description was applied to both acute and convalescent samples independently. | 10 |
| --- | --- |

**X.2 Prior Specification**

We use the following priors: $\Theta$~(0,1), $\Lambda$~𝑁(0,2) and, 𝛽~𝑈𝑛𝑖𝑓𝑜𝑟𝑚(−0.75,0.75). Variance parameters $\sigma_{\theta}$ 𝑎𝑛𝑑 $\sigma_{\alpha}$ follow zero-truncated standard normal distributions. We allow prevalence and specificity of the index test in each study to be uniform over 0 to 1. The sensitivity of the index test in each study are also assumed uniform between a lower limit of 1 minus the specificity. This ensures that the probability of a positive test is higher for somebody with disease than without.

In both analyses, the comparator tests are Culture and PCR. For culture, the sensitivity is assumed uniform between 0 and 1 however the specificity is assumed to follow a $beta(50,1)$ distribution. Assuming a $beta(50,1)$ distribution corresponds to an assumption of 95% probability of the specificity being above 94%. We assume the sensitivity and specificity of PCR follow a $beta(5,1)$ distribution. This corresponds to an assumption of 95% probability that the sensitivity and specificity of PCR are above 50%.

**X.3 Sensitivity Analysis**

Sensitivity analyses were conducted to assess the robustness of our results to assumptions about conditional dependence between tests and to the endemicity level of countries. In the main text of the article a model relaxing the assumption of conditional independence between diagnostic tests is presented. Here we present the results assuming conditional independence, comparing to those presented in the main text. We also present the results when only those studies from endemic studies are included. Table 1 highlights that the pooled and predicted estimates in acute samples did not differ greatly between models. Table 2 shows that in convalescent samples only including endemic countries slightly increased the median estimate of pooled and predicted sensitivity and specificities but that there is little evidence of conditional dependence between tests as results comparing whether conditional independence is assumed or relaxed are similar.

**Table S1: Sensitivity analysis in acute samples**

| **Study countries included** | **Conditional independence (CI) or conditional dependence (CD)** | **Pooled sensitivity median (95% CrI)** | **Pooled specificity median (95% CrI)** | **Predicted sensitivity median (95% CrI)** | **Predicted specificity median (95% CrI)** |
| --- | --- | --- | --- | --- | --- |
| All | CI | 17(5-41) | 88(66-98) | 17(1-89) | 89(14-100) |
| All* | CD | 14(3-38) | 86(59-96) | 14(0-90) | 86(9-100) |
| Endemic only | CI | 13(3-37) | 90(67-98) | 13(0-86) | 90(14-100) |
| Endemic only | CD | 11(2-33) | 87(60-97) | 10(0-86) | 88(10-100) |

*This row represents the results presented in the main text of the manuscript

**Table S2: Sensitivity analysis in convalescent samples**

| **Study countries included** | **Conditional independence (CI) or conditional dependence (CD)** | **Pooled sensitivity median (95% CrI)** | **Pooled specificity median (95% CrI)** | **Predicted sensitivity median (95% CrI)** | **Predicted specificity median (95% CrI)** |
| --- | --- | --- | --- | --- | --- |
| All | CI | 70(32-92) | 74(43-93) | 70(2-100) | 76(2-100) |
| All* | CD | 68(32-92) | 75(45-93) | 69(2-100) | 75(2-100) |
| Endemic only | CI | 74(38-95) | 81(51-95) | 76(2-100) | 81(4-100) |
| Endemic only | CD | 73(34-95) | 81(48-95) | 73(2-100) | 81(3-100) |

*This row represents the results presented in the main text of the manuscript

**References:**

1. Dendukuri N, Joseph L. Bayesian approaches to modeling the conditional dependence between multiple diagnostic tests. Biometrics. 2001;57(1):158-67.
2. Rutter CM, Gatsonis CA. A hierarchical regression approach to meta-analysis of diagnostic test accuracy evaluations. Stat Med. 2001;20(19):2865-84.
3. Dendukuri N, Schiller I, Joseph L, Pai M. Bayesian meta-analysis of the accuracy of a test for tuberculous pleuritis in the absence of a gold standard reference. Biometrics. 2012;68(4):1285-93.
