# Supplementary material for: Diagnosis of human leptospirosis: systematic review and meta-analysis of the diagnostic accuracy of the Leptospira microscopic agglutination test, PCR targeting Lfb1, and IgM ELISA to Leptospira fainei serovar Hurstbridge
Source: BMC Infect Dis. 2024 Feb 7;24:168. doi: 10.1186/s12879-023-08935-0 (PMC10848445; doi:10.1186/s12879-023-08935-0)
Supplement: Supplementary file 3 — Additional file 3: Appendix S3. List of studies excluded dued to not having enough data available for a 2x2 contingent table for single-acute phase samples and/or paired samples. [file 12879_2023_8935_MOESM3_ESM.docx]

**Appendix S3: List of studies excluded dued to not having enough data available for a 2x2 contingent table for single-acute phase samples and/or paired samples.**

1) De-Abreu-Fonseca C, Teixeira-de-Freitas VL, Caló-Romero E, Spinosa C, Arroyo-Sanches MC, da-Silva MV, Shikanai-Yasuda MA: Polymerase chain reaction in comparison with serological tests for early diagnosis of human leptospirosis. Trop Med Int Health 2006, 11:11.

2) Ooteman MC, Vago AR, Koury MC: Evaluation of MAT, IgM ELISA and PCR methods for the diagnosis of human leptospirosis. J Microbiol Methods 2006, 65:2.

3) Mendoza MT, Roxas EA, Ginete JK, Alejandria MM, Roman AD, Leyritana KT, Penamora MA, Pineda CC: Clinical profile of patients diagnosed with leptospirosis after a typhoon: a multicenter study. Southeast Asian J Trop Med Public Health 2013, 44:6.

4) Pérez-García J, Agudelo-Flórez P, Parra-Henao GJ, Ochoa JE, Arboleda M: Incidence and underreporting of leptospirosis comparing three diagnostic methods in the endemic region of Urabá, Colombia. Biomedica 2019, 39:1.

5) Wuthiekanun V, Sirisukkarn N, Daengsupa P, Sakaraserane P, Sangkakam A, Chierakul W, Smythe LD, Symonds ML, Dohnt MF, Slack AT, Day NP, Peacock SJ: Clinical diagnosis and geographic distribution of leptospirosis, Thailand. Emerg Infect Dis 2007, 13:1.

6) Doungchawee G, Sutdan D, Niwatayakul K, Inwisai T, Sitthipunya A, Boonsathorn N, Sakulterdkiat T, Sirawaraporn W, Thongboonkerd V. Development and evaluation of an immunochromatographic assay to detect serum anti-leptospiral lipopolysaccharide IgM in acute leptospirosis. Sci Rep 2017, 7:1.

7) Thaipadungpanit J, Chierakul W, Wuthiekanun V, Limmathurotsakul D, Amornchai P, Boonslip S, Smythe LD, Limpaiboon R, Hoffmaster AR, Day NP, Peacock SJ: Diagnostic accuracy of real-time PCR assays targeting 16S rRNA and lipL32 genes for human leptospirosis in Thailand: a case-control study. PLoS One 2011, 6:1.

8) Phimda K, Hoontrakul S, Suttinont C, Chareonwat S, Losuwanaluk K, Chueasuwanchai S, Chierakul W, Suwancharoen D, Silpasakorn S, Saisongkorh W, Peacock SJ, Day NP, Suputtamongkol Y: Doxycycline versus azithromycin for treatment of leptospirosis and scrub typhus. Antimicrob Agents Chemother 2007, 51:9.

9) Waggoner JJ, Balassiano I, Mohamed-Hadley A, Vital-Brazil JM, Sahoo MK, Pinsky BA: Reverse-Transcriptase PCR Detection of Leptospira: Absence of Agreement with Single-Specimen Microscopic Agglutination Testing. PLoS One 2015, 10:7.

10) Wuthiekanun V, Chierakul W, Limmathurotsakul D, Smythe LD, Symonds ML, Dohnt MF, Slack AT, Limpaiboon R, Suputtamongkol Y, White NJ, Day NP, Peacock SJ: Optimization of culture of Leptospira from humans with leptospirosis. J Clin Microbiol 2007, 45:4.

11) Cardona MN, Moros RM, López EA, Pérez JL, Hernández RC: Diagnóstico de leptospirosis mediante la PCR en pacientes con síndrome febril icterohemorrágico. Rev. Soc. Ven. Microbiol 2008, 28:1.

12) Kitashoji E, Koizumi N, Lacuesta TL, Usuda D, Ribo MR, Tria ES, Go WS, Kojiro M, Parry CM, Dimaano EM, Villarama JB, Ohnishi M, Suzuki M, Ariyoshi K: Diagnostic Accuracy of Recombinant Immunoglobulin-like Protein A-Based IgM ELISA for the Early Diagnosis of Leptospirosis in the Philippines. PLoS Negl Trop Dis 2015, 9:6.

13) Mohit B, Umapathy BL, Navaneeth BV: Evaluation Of Diagnostic Utility Of Modified Faine’s Criteria In Leptospirosis- Experience From A Tertiary Care Hospital. NJIRM 2015, 6:4.

14) Neela VK, Philip N, Sekawi Z: Leptospirosis: Malaysia Leptospirosis Research network experience. International Journal of Infectious Diseases 2020, 101:1.

15) Ko AI, Galvão Reis M, Ribeiro Dourado CM, Johnson WD Jr, Riley LW. Urban epidemic of severe leptospirosis in Brazil. Salvador Leptospirosis Study Group. Lancet 1999, 354:9181.

16) Agampodi SB, Dahanayaka NJ, Nöckler K, Mayer-Scholl A, Vinetz JM. Redefining Gold Standard Testing for Diagnosing Leptospirosis: Further Evidence from a Well-Characterized, Flood-Related Outbreak in Sri Lanka. Am J Trop Med Hyg 2016, 95:3.

17) Yuhana MY, Hanboonkunupakarn B, Tanganuchitcharnchai A, Sujariyakul P, Sonthayanon P, Chotivanich K, Pukrittayakamee S, Blacksell SD, Paris DH: Rickettsial Infections Are Neglected Causes of Acute Febrile Illness in Teluk Intan, Peninsular Malaysia. Trop Med Infect Dis 2022, 7:5.

18) Merien F, Baranton G, Perolat P: Comparison of Polymerase Chain Reaction with Microagglutination Test and Culture for Diagnosis of Leptospirosis. The Journal of Infectious Diseases 1995; 172:281-5.

19) Céspedes M, Tapia R, Balda L, Gonzalez D, Peralta C, Condori P: Estandarización y validación de una prueba de PCR para el diagnóstico precoz de leptospirosis humana. Rev Peru Med Exp Salud Publica 2007, 24:1.

20) Céspedes M, Tapia R, Balda L, Gonzalez D, Glenny M, Vinetz JM: Brote de leptospirosis asociado a la natación en una fuente de agua subterránea en una zona costera, Lima-Perú. Rev Peru Med Exp Salud Publica 2009, 26:4.

21) Goris MGA, Leeflang MMG, Loden M, Wagenaar JFP, Klatser PR, et al: Prospective Evaluation of Three Rapid Diagnostic Tests for Diagnosis of Human Leptospirosis. PLoS Negl Trop Dis 2013, 7:7.

22) Effler P, Domen HY, Bragg SL, Aye T, Sasaki DM: Evaluation of the Indirect Hemagglutination Assay for Diagnosis of Acute Leptospirosis in Hawaii. Journal of Clinical Microbiology 2000, 38:3.

23) Chaudhry R, Das A, Premlatha MM, Choudhary A, Chourasia BK, Chandel DS, Dey AB: Serological & molecular approaches for diagnosis of leptospirosis in a tertiary care hospital in north India: A 10-year study. Indian J Med Res 2013, 137:4.

24) Warnasekara J, Srimantha S, Kappagoda C, Jayasundara D, Senevirathna I, Matthias M, Agampodi S, Vinetz JM: Diagnostic method-based underestimation of leptospirosis in clinical and research settings; an experience from a large prospective study in a high endemic setting. PLoS Negl Trop Dis 2022, 16:4.

25) Cumberland P, Everard COR, Levett PN: Assessment of the efficacy of an IgM-ELISA and microcopic agglutination test (MAT) in the diagnosis of acute leptospirosis. American Journal of Tropical Medicine and Hygiene 1999, 61:5.

26) Goris MG, Leeflang MM, Boer KR, Goeijenbier M, van Gorp EC, Wagenaar JF, et al. Establishment of valid laboratory case definition for human leptospirosis. J Bacteriol Parasitol 2012, 3:1000132.
