# Supplementary material for: Diagnosis of human leptospirosis: systematic review and meta-analysis of the diagnostic accuracy of the Leptospira microscopic agglutination test, PCR targeting Lfb1, and IgM ELISA to Leptospira fainei serovar Hurstbridge
Source: BMC Infect Dis. 2024 Feb 7;24:168. doi: 10.1186/s12879-023-08935-0 (PMC10848445; doi:10.1186/s12879-023-08935-0)
Supplement: Supplementary file 4 — Additional file 4: Table S3. Standardized extraction sheet form in the systematic review of studies evaluating the diagnostic accuracy of MAT, PCR, and IgM ELISA, published global and between 1950–2022. [file 12879_2023_8935_MOESM4_ESM.docx]

**Table S3:** **Standardized extraction sheet form in the systematic review of studies evaluating the diagnostic accuracy of MAT, PCR, and IgM ELISA, published global and between 1950 – 2022.**

General features: Study number, First author, Year, Country

Study characteristics:

Type of study

Recruitment setting

Inclusion criteria

Exclusion criteria

Participant’s age and gender

Participant’s fever/symptoms/suspected disease

MAT panel and case definition

Referent tests

Results for each reference test:

Number of patients included: True positive, False positive, False negative, True negative.

Number single acute-phase samples: True positive, False positive, False negative, True negative.

Number paired samples: True positive, False positive, False negative, True negative.
