# Supplementary material for: Diagnosis of human leptospirosis: systematic review and meta-analysis of the diagnostic accuracy of the Leptospira microscopic agglutination test, PCR targeting Lfb1, and IgM ELISA to Leptospira fainei serovar Hurstbridge
Source: BMC Infect Dis. 2024 Feb 7;24:168. doi: 10.1186/s12879-023-08935-0 (PMC10848445; doi:10.1186/s12879-023-08935-0)
Supplement: Supplementary file 5 — Additional file 5: Table S4. Extracted data in the systematic review of studies evaluating the diagnostic accuracy of MAT, published global and between 1950–2022. [file 12879_2023_8935_MOESM5_ESM.docx]

**Table S4: Extracted data in the systematic review of studies evaluating the diagnostic accuracy of MAT, published global and between 1950 – 2022.**

S4 - Endemic countries

Single acute-phase samples - Reference test Blood culture

| **Study first author, ref** | **Reference test culture** | **Total N samples** | **Index+/Reference+** | **Index+/Reference-** | **Index-/Reference+** | **Index-/Reference-** |
| --- | --- | --- | --- | --- | --- | --- |
| Woods K (25) | Culture | 787 | 0 | 20 | 4 | 763 |
| Dittrich S (28) | Culture | 695 | 1 | 46 | 3 | 645 |
| Albuquerque (29) | Culture | 12 | 1 | 6 | 1 | 4 |
| Dinhuzen J (31) | Culture | 99 | 0 | 5 | 5 | 89 |
| Sukmark T (36) | Culture | 211 | 7 | 70 | 20 | 114 |

Single acute-phase samples - Reference test PCR

| **Study first author, ref** | **Reference test PCR** | **Total N samples** | **Index+/Reference+** | **Index+/Reference-** | **Index-/Reference+** | **Index-/Reference-** |
| --- | --- | --- | --- | --- | --- | --- |
| Woods K (25) | PCR rrs | 787 | 0 | 20 | 14 | 753 |
|  | PCR PHE | 787 | 0 | 20 | 11 | 756 |
| Blanco (26) | PCR | 521 | 0 | 2 | 4 | 515 |
|  | Nested PCR | 521 | 0 | 2 | 24 | 495 |
| Merien F (27) | PCR rrs | 51 | 0 | 1 | 15 | 35 |
| Dittrich S (28) | PCR | 687 | 1 | 46 | 12 | 628 |
| Philip N (30) | PCR rrs | 165 | 11 | 16 | 52 | 86 |
|  | PCR LipL32 | 165 | 11 | 16 | 36 | 102 |
| Dinhuzen J (31) | PCR | 99 | 4 | 1 | 48 | 46 |
| Alia SN (35) | PCR | 50 | 0 | 6 | 13 | 31 |

Paired samples – Reference test Blood culture

| **Study first author, ref** | **Reference test culture** | **Total N samples** | **Index+/Reference+** | **Index+/Reference-** | **Index-/Reference+** | **Index-/Reference-** |
| --- | --- | --- | --- | --- | --- | --- |
| Woods K (25) | Culture | 238 | 1 | 9 | 3 | 225 |
| Dittrich S (28) | Culture | 248 | 1 | 9 | 3 | 235 |
| Albuquerque (29) | Culture | 9 | 1 | 4 | 0 | 4 |
| Dinhuzen J (31) | Culture | 72 | 4 | 22 | 0 | 46 |
| Mullan S (32) | Culture | 207 | 0 | 161 | 0 | 46 |
| Vijayachari P (33) | Culture | 124 | 0 | 40 | 34 | 50 |
| Kakita T (34) | Culture | 198 | 72 | 51 | 0 | 75 |

Paired samples – Reference test PCR

| **Study first author, ref** | **Reference test PCR** | **Total N samples** | **Index+/Reference+** | **Index+/Reference-** | **Index-/Reference+** | **Index-/Reference-** |
| --- | --- | --- | --- | --- | --- | --- |
| Woods K (25) | PCR rrs | 238 | 1 | 9 | 14 | 214 |
|  | PCR PHE | 238 | 1 | 9 | 11 | 217 |
| Blanco (26) | PCR | 521 | 4 | 24 | 0 | 493 |
|  | Nested PCR | 521 | 24 | 4 | 0 | 493 |
| Merien F (27) | PCR rrs | 10 | 10 | 0 | 0 | 0 |
| Dittrich S (28) | PCR | 248 | 0 | 10 | 13 | 225 |
| Philip N (30) | PCR rrs | 63 | 3 | 13 | 8 | 39 |
|  | PCR LipL32 | 63 | 3 | 13 | 5 | 42 |
| Dinhuzen J (31) | PCR | 49 | 8 | 5 | 15 | 21 |
| Kakita T (34) | PCR | 268 | 51 | 46 | 35 | 136 |

S4 – non-endemic countries

Single acute-phase samples - Reference test Blood culture

| **Study first author, ref** | **Reference test culture** | **Total N samples** | **Index+/Reference+** | **Index+/Reference-** | **Index-/Reference+** | **Index-/Reference-** |
| --- | --- | --- | --- | --- | --- | --- |
| Podgoršek D (37) | Culture | 24 | 4 | 9 | 2 | 9 |
| Podgoršek D (38) | Culture | 10 | 1 | 2 | 1 | 6 |

Single acute samples - Reference test PCR

| **Study first author, ref** | **Reference test PCR** | **Total N samples** | **Index+/Reference+** | **Index+/Reference-** | **Index-/Reference+** | **Index-/Reference-** |
| --- | --- | --- | --- | --- | --- | --- |
| Podgoršek D (38) | PCR | 20 | 3 | 1 | 5 | 11 |

Paired samples - Reference test Blood culture

| **Study first author, ref** | **Reference test culture** | **Total N samples** | **Index+/Reference+** | **Index+/Reference-** | **Index-/Reference+** | **Index-/Reference-** |
| --- | --- | --- | --- | --- | --- | --- |
| Podgoršek D (38) | Culture | 9 | 1 | 7 | 1 | 0 |
| Earl L (39) | Culture Epi | 22 | 0 | 6 | 2 | 14 |
|  | Culture ESR | 22 | 1 | 5 | 1 | 15 |

Paired samples - Reference test PCR

| **Study first author, ref** | **Reference test PCR** | **Total N samples** | **Index+/Reference+** | **Index+/Reference-** | **Index-/Reference+** | **Index-/Reference-** |
| --- | --- | --- | --- | --- | --- | --- |
| Podgoršek D (38) | PCR | 14 | 5 | 8 | 0 | 1 |
| Earl L (39) | PCR Epi | 22 | 3 | 3 | 2 | 14 |
|  | PCR ESR | 22 | 1 | 5 | 1 | 15 |
|  | PCR CHL | 22 | 2 | 4 | 1 | 15 |
